# Low ADAMTS13 Activity Correlates with Increased Mortality in COVID-19 Patients

**DOI:** 10.1055/s-0041-1723784

**Published:** 2021-03-09

**Authors:** Joseph M. Sweeney, Mohammad Barouqa, Gregory J. Krause, Jesus D. Gonzalez-Lugo, Shafia Rahman, Morayma Reyes Gil

**Affiliations:** 1 Department Physiology and Biophysics, Albert Einstein College of Medicine, Bronx, New York, United States; 2 Department of Pathology Montefiore Medical Center, Albert Einstein College of Medicine, Bronx, New York, United States; 3 Department of Developmental and Molecular Biology, Albert Einstein College of Medicine, Bronx, New York, United States; 4 Institute of Aging Studies, Albert Einstein College of Medicine, Bronx, New York, United States; 5 Division of Hematology, Department of Medical Oncology, Albert Einstein College of Medicine and Montefiore Medical Center, Bronx, New York, United States

**Keywords:** von Willebrand factor, ADAMTS13, COVID-19, schistocytes, coagulopathy

## Abstract

The causes of coagulopathy associated with coronavirus disease 2019 (COVID-19) are poorly understood. We aimed to investigate the relationship between von Willebrand factor (VWF) biomarkers, intravascular hemolysis, coagulation, and organ damage in COVID-19 patients and study their association with disease severity and mortality. We conducted a retrospective study of 181 hospitalized COVID-19 patients randomly selected with balanced distribution of survivors and nonsurvivors. Patients who died had significantly lower ADAMTS13 (a disintegrin and metalloproteinase with a thrombospondin type 1 motif, member 13) activity, significantly elevated lactate dehydrogenase levels, significantly increased shistocyte/RBC fragment counts, and significantly elevated VWF antigen and activity levels compared with patients discharged alive. These biomarkers correlate with markedly elevated D-dimers. Additionally, only 30% of patients who had an ADAMTS13 activity level of less than 43% on admission survived, yet 60% of patients survived who had an ADAMTS13 activity level of greater than 43% on admission. In conclusion, COVID-19 may present with low ADAMTS13 activity in a subset of hospitalized patients. Presence of schistocytes/RBC fragment and elevated D-dimer on admission may warrant a work-up for ADAMTS13 activity and VWF antigen and activity levels. These findings indicate the need for future investigation to study the relationship between endothelial and coagulation activation and the efficacy of treatments aimed at prevention and/or amelioration of microangiopathy in COVID-19.

## Introduction

Coronavirus disease 2019 (COVID-19) is a respiratory disease with heterogeneous manifestations ranging from asymptomatic illness in some, to systemic inflammation, multiorgan failure, and a rapid death in others. 1 2 The first stage of disease manifests as an upper respiratory infection followed by pneumonia when the virus invades the respiratory epithelium via binding to angiotensin converting enzyme 2 (ACE2) receptors. 3 A second, more severe, phase may be manifested as multiorgan damage, including respiratory, cardiac, hepatic, and renal injury. At this stage, the ACE2 receptors on the endothelium can also be involved, causing direct damage to blood vessels and inducing a coagulopathy. 3

Systemic inflammation and coagulopathy are characteristic hallmarks of this phase. “COVID coagulopathy” manifests mainly as a prothrombotic state affecting both large and small blood vessels, and presenting as arterial, venous, and microangiopathic thrombotic events. 4 5 Coagulopathy with D-dimer elevations is reported in most hospitalized COVID-19 patients. 6 7 8 A recent study showed that markers of endothelial damage such as von Willebrand factor (VWF) and soluble thrombomodulin were also increased in COVID-19 hospitalized patients. All these markers were even higher in intensive care unit patients and correlated with mortality. 9 VWF has three main functions: binding to collagen in the wounded subendothelial matrix, binding to glycoprotein-1b on platelets, and carrying then subsequently delivering coagulation factor VIII (FVIII) to the surface of activated platelets bound to wounded endothelium. 10 Whether the increased VWF reported in COVID-19 is a result of increased production, abnormal and/or increased release, or decreased destruction is unclear. Since ADAMTS13 (a disintegrin and metalloproteinase with a thrombospondin type 1 motif, member 13), a VWF-cleaving protease, plays a key role in regulating both VWF quantity and multimer size, analysis of this enzyme would be important in elucidating the pathophysiology of COVID coagulopathy. Although there have been some COVID-19 data involving ADAMTS13 activity levels, the small sample size in these reports precluded any major conclusions. 11 12 13

The primary objective of our study was to establish the relationship of VWF-related biomarkers with coagulation, thrombosis, intravascular hemolysis, and end-organ damage in a large cohort of COVID hospitalized patients. The secondary objective was to study the correlation of VWF-related biomarkers with disease severity and mortality.

## Methods

### Study Population

We included confirmed COVID-19 cases in Montefiore Medical Center who were hospitalized and had routine and/or advanced coagulation tests done between March 26, 2020 and May 5, 2020. All included patients tested positive for SARS-CoV2 with reverse-transcriptase–polymerase-chain-reaction real-time assay of the nasal and the pharyngeal swabs. We excluded the patients younger than 18 years of age. The medical records of the patients were reviewed to obtain epidemiological, demographic, clinical, and laboratory data. The management and clinical outcomes were followed-up until June 20, 2020. All cases had final disposition (deceased or discharged alive) and none were censored. The study was approved by the Albert Einstein College of Medicine Institutional Review Board.

### Laboratory Investigations

A total of 3,672 plasma samples were aliquoted from sodium citrate tubes shortly after blood draw and stored at −80°C until needed. Samples were deidentified, coded with a unique arbitrary number, sorted by this arbitrary number (lowest to highest), and grouped into two categories: deceased versus discharged alive cases. To study the association of disease severity with VWF antigen, VWF activity, and ADAMTS13 activity, samples were then selected down the list (top to bottom) with balanced number of discharged alive versus deceased patients across the range of D-dimer levels from normal to very high (0.26 to >20 μg/mL) ( *n* = 128). These cases were stratified by D-dimer categories: low (<2 μg/mL), moderate (2–10 μg/mL), and high (>10 μg/mL) (study design chart in Supplementary Fig. S1 ). To study if initial ADAMTS13 activity at presentation has predictive value, we selected an additional 40 cases with a sample collected within 72 hours since admission. This selection, like the original selection, was random going down the list with similar numbers of discharged alive and deceased patients. To study the association between platelet count and ADAMTS13 activity, we selected 13 patients who had a platelet count < 70 × 10 ^6^ /μL. This platelet count was predefined, as it is a common cut off to differentiate mild from moderate thrombocytopenia in our institute. Altogether the total number of cases was 181 (study design chart in Supplementary Fig. S1 ).

### Statistical Analysis

Data analysis was performed using R studio, V.3.6.2 and graphs generated in Prism V.8.3.1. Differences in demographic, clinical variables, and laboratory assessments between patients who were deceased, and patients discharged alive were compared using Chi-square or Fisher's exact tests for categorical variables, two-sample Student *t* -tests, and one-way ANOVA for three group comparisons. Youden's *J* statistics was used to find the optimal cut point for ADAMTS13 activity for mortality. Logistic regression of initial ADAMTS13 activity adjusted by age was represented in a density plot against mortality. Logistic regression was also used to adjust BMI by age. A Kaplan-Meier cumulative curve was generated for patients discharged alive.

#### Laboratory Testing

Coagulation tests (VWF Antigen, VWF Ristocetin activity [VWF:RCo], FVIII activity levels, D-dimer, and fibrin monomer [FM]) were performed by STA-R Max instruments using STAGO reagents as per manufacturer recommendations. We validated the Siemens BC VWF Ristocetin cofactor reagents in the STA-R Max as previously described. 14 STA Liatest LIA D-dimer assay was performed as per manufacturer recommendations and reported as fibrinogen-equivalent-units (FEU) μg/mL. Complete blood counts (CBCs) were performed by Sysmex XN9000. Chemistry assays were performed by Roche instrumentation and reagents as per manufacturer recommendations.

After thawing the patient samples in a 37°C water bath, a 1:1 dilution was created using patient plasma and STAGO Owren-Koller buffer. Using this dilution, VWF antigen, VWF:RCo, FVIII activity levels, D-dimer, and FM levels were obtained. Our reportable range for VWF antigen is 420%, VWF activity 650%, and FVIII activity is 600%. If the STA-R Max instrument was not able to accurately report the value due to it being above the reportable range, a 1:20 dilution was made with Owren-Koller buffer and subsequently a 1:100 dilution until the value was within reportable range. All results were multiplied by the dilution factor to obtain the final value.

#### Data Gathering

Chart reviews were performed to document demographic attributes (age, sex, and self-reported race and/or ethnicity) and baseline comorbidities (body mass index, previous history of hypertension, diabetes, kidney, pulmonary, liver, autoimmune, cancer, or sickle cell disease) on presentation collected for calculation of comorbidity indexes. We gathered data on initial vital signs and laboratory values within the first 48 hours of hospital admission. The laboratory assessments consisted of a CBC, blood chemical analysis, coagulation testing, assessment of liver and renal function, measures of electrolytes, and markers of inflammation. Additionally, we noted the D-dimer, fibrinogen, hemoglobin, creatine, lactate dehydrogenase (LDH), indirect bilirubin, and platelet count of the patients within 48 hours of the time of collection of the samples we tested. We also noted the trajectory of these parameters in the week following the collection of the sample, noting whether these parameters increased by 10% or more, decreased by 10% or more, or remained stable. We accessed each patient for thrombosis and clot formation. A patient was considered to have thrombosis if a thrombus was identified on radiological imaging. We also noted if a patient experienced ex vivo clotting while on hemodialysis (HD) or continuous renal replacement therapy based on the need for kit/filter change and/or visual clots as documented in the clinical progress notes. We documented anticoagulation medications given to each patient within 48 hours preceding the thrombus or clotting event.

#### STROBE Criteria

This study followed the STROBE criteria for retrospective studies including: (1) providing a summary in the abstract of the objectives, the study type, outcome, and conclusion; (2) providing scientific background, rationale, and hypothesis in the introduction; (3) providing details of the study design in the methods including setting, participants, sample size, variables, data sources, and measurements; (4) providing details of statistical methods; (5) describing demographics of the population in the results; (6) providing 95% confidence intervals in the results when appropriate; (7) providing a discussion of the limitations, potential bias, and generalizability.

#### VWF Multimers

VWF multimers were generated using a western blot technique previously described. 15 Briefly, samples were first prepared by normalizing the amount of thawed plasma added to the loading dye according to the measured VWF antigen level. The samples were then heat inactivated for at least 15 minutes in a 56°C water bath. The samples subsequently were loaded onto a 1% agarose gel. A normal control consisting of normal pooled plasma and an abnormal control consisting of plasma from a patient with Type 2A Von Willebrand disease were used. The gels were run using the PhastSystem Separation and Control unit. After this, the gels were transferred to a polyvinylidene difluoride membrane also using the PhastSystem Separation and Control unit. After the transfer, the membranes were blocked for at least 40 minutes using 1.2% bovine serum albumin (BSA) (Sigma-Aldrich) in tris-buffered saline plus 0.04% Tween 20. After blocking, rabbit anti-human F. VIII-related antigen antibody (Accurate Chemical AXL 205) was applied as the primary antibody for 45 minutes at a concentration of 5.58 µg/mL diluted in 1.2% BSA. Anti-rabbit IgG-alkaline phosphatase-conjugated antibody (Sigma A8025) was applied as the secondary antibody for 45 minutes at a concentration of 1.5 µg/mL diluted in 1.2% BSA. After this, SigmaFast 5-Bromo-4-chloro-3-indolyl phosphate/nitro blue tetrazolium (BCIP/NBT) solution was used to develop the blot.

Western blots were then analyzed using the ImageJ analysis software. Briefly, each sample well was segmented into low, intermediate, and high molecular weight VWF multimers. Low molecular weight multimers were defined as the bottom three bands of the well. The division between intermediate and high molecular weight multimers (HMWMs) was established using the abnormal control that was run in each gel, as the abnormal control has no HMWMs. A straight line was drawn across the gel where the HMWM signal in the abnormal control started to taper. Intermediate size multimers were those between the cut-off established with the abnormal control and the highest band of the low molecular weight multimers. The measure function in ImageJ was used to measure the raw integrated density of each size of the multimers. All values were normalized to total VWF protein loaded per well. Values are reported as fold change from normal control.

#### VWF Collagen Binding Activity

Previously thawed plasma was centrifuged for 10 minutes at 24,328xg and the supernatant was used for the VWF collagen binding activity (VWF:CB) enzyme-linked immunosorbent assay (ELISA) and ADAMTS13 activity assay. Human VWF:CB was measured using Zymutest VWF:CB ELISA Kit (#RK038 from Hyphen BioMed). The ELISA was completed and analyzed using the manufacturer's recommendations.

#### ADAMTS13 Activity

The ADAMTS13 protease activity on previously thawed and centrifuged plasma supernatant was measured using ATS-13 activity assay based on fluorescence resonance energy transfer (Immucor ATS-13). The assay was performed and analyzed using the manufacturer's recommendations. For patients whose plasma samples had an ADAMTS13 activity level of less than 30%, we ran an inhibitor assay according to the manufacturer's recommendations. Briefly, this assay involved mixing equal volumes of normal pooled plasma with the patient's plasma and measuring the ADAMTS13 activity of the mixed sample relative to that of the normal pooled plasma to find the percent inhibition. We considered a value between 25 to 40% mild inhibition and greater than 40% inhibition to indicate a true inhibitor.

#### ADAMTS13 Antigen

The ADAMTS13 antigen level was measured on previously thawed and centrifuged plasma supernatant using Technozym ADAMTS13 Fluorogenic Activity/Antigen (cat# 5450551). The ELISA was completed and analyzed using the manufacturer's recommendations.

#### ADAMTS13 Antibody Detection

The presence of human IgG autoantibodies against ADAMTS13 was determined using Technozym ADAMTS13 Inhibitor ELISA (cat# 5450451). The ELISA was completed and analyzed using the manufacturer's recommendations.

#### Peripheral Blood Smear

CBCs were performed on admission and when clinically indicated during hospitalization. Manual differentials were performed when reflexed due to a count threshold or scattergram abnormality. We analyzed all the smears available in which a smear review was reflexed due to an abnormality in the white blood cells (WBCs), red blood cells (RBCs), platelet count, or scattergram. Smear photos were obtained from CellaVision. Schistocytes, RBC fragments, and ghost cell count were based on at least 1,000 RBCs. RBC count was performed by using the digital manual counter on Image J. We noticed that cases in which the schistocytes were less than 1%, the smear review was not prompted by RBC or platelet flags but were prompted by unrelated flags, e.g., WBC flags. As previously reported, the sensitivity of RBC or platelet flags to detect schistocytes/RBC fragments is less than 1% (0.6–0.9%). 16 Thus, we classified all cases with no RBC/platelet flags as <1% schistocytes/RBC fragments.

## Results

### Study Population

Samples from 90 patients who died and 91 who were discharged alive with a wide and balanced distribution of D-dimer levels (0.26 to >20 μg/mL) were selected ( Supplementary Fig. S1 ). The characteristics of the study population are summarized in Table 1 . Consistent with many other studies, nonsurvivors were older (median [interquartile range or IQR]; 72.5 [63.3, 79.8] vs. 62.0 [50.5, 70.0] years) and the majority were males (67% [60/90], *p* = 0.03) ( Table 1 ). No difference between survivors and nonsurvivors by ethnicity or comorbidity was observed. Although the median BMI was higher in survivors, when BMI was adjusted by age, there was no significant difference between survivors and nonsurvivors ( *p* = 0.54, data not shown). As expected, the number of patients that required a ventilator was higher in nonsurvivors 51% (46/90) versus 26% (24/91) in survivors. No significant difference in the average length of stay or treatment was observed.

**Table 1:** Characteristics and initial clinical laboratory data of 181 patients with COVID-19 stratified by mortality outcome

Characteristics		Discharged alive ( *n* = 91) (median [IQR] or *n* (%))	Deceased ( *n* = 90) (median [IQR] or *n* (%))	*p* -Value
Age		62.0 [50.5, 70.0]	72.5 [63.3, 79.8]	<0.001
Sex (male)		46 (51)	60 (67)	0.03
Race	Black or African-American	36 (40)	34 (38)	0.81
	White	9 (10)	11 (12)	0.81
	Other/Patient declined/not reported	46 (51)	44 (49)	0.82
Ethnicity	Spanish/Hispanic/Latino	36 (40)	33 (37)	0.69
BMI (kg/m ^2^ )		30.1 [27.2, 34.0]	28.5 [25.5, 31.8]	0.04
Elixhauser comorbidity index		4.0 [1.0, 6.3]	5.0 [2.0, 8.0]	0.14
Length of stay (days)		9.0 [5.0, 27.0]	10.5 [5.3, 16.0]	0.73
Date of sample after admission (days)		3.0 [1.0, 6.5]	3.0 [1.0, 8.0]	0.75
In vivo thrombosis or ex vivo clot		23 (25)	20 (22)	0.63
Invasive ventilator use		24 (26)	46 (51)	<0.001
Vasopressor use		21 (23)	31 (34)	0.09
Hemodialysis or CRRT use		17 (19)	23 (26)	0.27
Anticoagulation use a		34 (37)	33 (37)	0.92
Steroid use b		29 (32)	31 (34)	0.71
**Initial clinical laboratory values measured upon admission (units) [reference range]**				
eGFR (mL/min/1.73 m ^2^ ) [90–120]		74.5 [34.3, 98.8]	40.5 [25.6, 70.8]	<0.001
Creatinine (mg/dL) [0.84–1.21]		1.0 [0.7, 1.9]	1.7 [1.1, 2.5]	<0.001
Anion gap (mEq/L) [8–16]		17.0 [15.0, 20.0]	18.0 [16.0, 22.0]	0.03
Aspartate transaminase (IU/L) [8–48]		44.0 [28.3, 68.0]	51.0 [37.0, 78.8]	0.08
Hemoglobin (g/dL) [12–17.5]		12.9 [11.5, 13.8]	13.0 [10.8, 14.2]	0.63
Mean corpuscular volume (fL) [80–95]		89.9 [85.2, 92.9]	89.8 [84.2, 96.0]	0.64
White blood cell (10 ^3^ /µL) [4.8–10.8]		6.8 [5.1, 10.0]	7.3 [5.5, 11.4]	0.21
Neutrophil to lymphocyte ratio [0.78–3.53]		5.9 [3.0, 9.0]	7.1 [4.6, 11.3]	0.02
Platelet (Count) (k/µL) [150–450]		213.0 [157.3, 288.0]	183.0 [136.0, 270.0]	0.14
Mean platelet volume (fL) [7–12]		10.8 [10.1, 11.8]	11.1 [10.4, 11.9]	0.18
D-dimer (µg/mL) [<0.27]		1.8 [0.7, 3.9]	2.6 [1.3, 5.7]	0.03
International normalized ratio [<1.1]		1.1 [1.0, 1.2]	1.1 [1.0, 1.3]	0.65
C-reactive protein (mg/L) [<10]		12.4 [4.9, 20.3]	15.9 [6.9, 26.1]	0.06
Lactate dehydrogenase (U/L) [140–280]		451.0 [277.0, 658.0]	542.0 [391.0, 652.0]	0.03
Troponin (ng/mL) [<0.04]		0.01 [0.01, 0.02]	0.03 [0.01, 0.06]	<0.001
Pulse oximeter (%) [95–100]		96.0 [91.0, 99.0]	94.0 [87.3, 97.8]	0.04
Diastolic blood pressure (mm Hg) [60–80]		75.0 [66.0, 85.0]	62.0 [42.8, 75.0]	<0.001

Abbreviations: BMI, body mass index; CRRT, continuous renal replacement therapies; eGFR, estimated glomerular filtration rate; IQR, interquartile range.

a Within 48 h prior to clot or ADAMTS13 activity measurement.

b Within 24 h prior to ADAMTS13 activity measurement.

### Initial Clinical Laboratory Data

Initial markers of renal function were significantly worse in nonsurvivors compared with survivors (creatinine, 1.7 [1.1, 2.5] vs. 1.0 [0.7, 1.9] mg/dL, *p* < 0.001) whereas markers of liver function were not significantly different. Oxygen saturation was lower in nonsurvivors compared with survivors (94.0 [87.3, 97.8] vs. 96.0 [91.0, 99.0]%; *p* = 0.04). Also, initial diastolic blood pressure was significantly lower in nonsurvivors versus survivors (62.0 [42.8, 75.0] vs. 75.0 [66.0, 85.0] mm Hg; *p* < 0.001). CBC parameters were not significantly different with the exception of neutrophil to lymphocyte ratio, (7.1 [4.6, 11.3] in nonsurvivors vs. 5.9 [3.0, 9.0] in survivors; *p* = 0.02).

The only hemolysis marker that was significantly higher in nonsurvivors was LDH (542.0 [391.0, 652.0] vs. 451.0 [277.0, 658.0] U/L; *p* < 0.001). Initial D-dimer was significantly higher in nonsurvivors (2.6 [1.3, 5.7] vs. 1.8 [0.7, 3.9] μg/mL; *p* = 0.03) ( Table 1 ).

### Correlation of VWF Antigen and Activity, ADAMTS13 Activity, and Coagulation Markers

We analyzed ADAMTS13 activity , VWF antigen and activity, FVIII activity, D-dimer, and FM concentration, a precursor of D-dimer concentration, on the same samples irrespective of the time since admission. Nonsurvivors had significantly lower ADAMTS13 activity levels (48.8 [36.2, 65.1] vs. 63.6 [47.2, 78.9]%; *p* ≤ 0.001) and higher FM (13.2 [5.0, 129.1] vs. 5.0 [5.0, 29.40]μg/mL; *p* = 0.02) and D-dimer levels (4.93 [1.83, 20.00] vs. 2.90 [0.92, 14.47]ug/mL; *p* = 0.04) than survivors ( Table 2 ). As expected, VWF antigen directly correlates with VWF:RCo ( *r* = 0.58; *p* ≤ 0.0001) and FVIII activity ( *r* = 0.34; *p* ≤ 0.001) ( Supplementary Fig. S2A, C ). Correspondingly, VWF:RCo correlates with VWF activity collagen binding (VWF:CB) ( *r* = 0.77; *p* ≤0.0001, Supplementary Fig. S2B ), although VWF:CB levels were proportionally higher than VWF:RCo levels, perhaps due to increased sensitivity of VWF:CB to HMWM. 17 Also as expected, ADAMTS13 activity inversely correlates with VWF:RCo ( *r* = −0.28; *p* = 0.0001) and VWF:CB ( *r* = −0.3; *p* = 0.009) ( Supplementary Fig. S2D and E ).

**Table 2:** ADAMTS13 activity levels and concurrent markers of endothelial activation, intravascular hemolysis, coagulation, and organ damage of 181 patients with COVID-19 stratified by mortality outcome

Clinical laboratory values (units) [reference range]	Discharged alive ( *n* = 91 a ) (median [IQR] or *n* (%))	Deceased ( *n* = 90 a ) (median [IQR] or *n* (%))	*p* -Value
ADAMTS13 activity (%) [70–110]	63.6 [47.2, 78.9]	48.8 [36.2, 65.1]	<0.001
Schistocyte/RBC fragment count (%) b [<0.5]	0.56 [0.16, 1.12] *n* = 31	1.06 [0.49, 2.63] *n* = 42	0.008
Schistocyte/RBC fragment b >1%	10 (11)	22 (24)	0.02
Lactate dehydrogenase (U/L) [140–280]	424.0 [275.0, 594.0]	562.5 [437.3, 664.8]	<0.001
Indirect bilirubin (mg/dL) [0.2–0.8]	0.2 [0.1, 0.4]	0.2 [0.1, 0.4]	0.89
Hemoglobin (g/dL) [12–17.5]	11.8 [10.1, 13.9]	11.7 [9.7, 13.7]	0.85
Platelet count (k/µL) [150–450]	241.0 [163.5, 344.5]	196.0 [124.8, 312.8]	0.06
Decreased platelet trajectory c	18 (20)	27 (30)	0.11
Creatinine (mg/dL) [0.84–1.21]	1.0 [0.7, 2.3]	1.9 [1.2, 3.5]	<0.001
Increased creatinine trajectory c	14 (15)	37 (41)	0.001
VWF activity (ristocetin) (%) [50–150]	282.0 [214.0, 400.0]	321.0 [238.0, 451.0]	0.05
VWF activity (collagen binding) (%) [50–150]	383.2 [235.2, 458.2] *n* = 35	368.7 [261.1, 585.2] *n* = 37	0.40
VWF antigen (%) [50–150]	362.0 [261.0, 540.0]	441.0 [307.6, 598.0]	0.05
D-dimer (µg/mL) [<0.27]	2.9 [0.9, 14.4]	4.93 [1.8, 20.0]	0.04
Fibrin monomer (µg/mL) [<10]	5.0 [5.0, 29.4]	13.2 [5.0, 129.1]	0.02
Factor VIII activity (%) [50–150]	175.0 [118.0, 247.5] *n* = 27	160.0 [106.5, 246.5] *n* = 58	0.46
HMW multimer fold change from normal d	0.95 [0.71, 1.25] *n* = 57	1.04 [0.83, 1.46] *n* = 58	0.14
Fibrinogen (mg/dL) [200–400]	572.0 [462.0, 727.0] *n* = 59	496.0 [376.3, 744.3] *n* = 50	0.27

Abbreviations: IQR, interquartile range; ADAMTS13, a disintegrin and metalloproteinase with a thrombospondin type 1 motif, member 13; VWF, Von Willebrand factor; HMW, high molecular weight.

a Unless otherwise stated.

b Within 3 days of ADAMTS13 activity testing.

c Based on a change >10% over 1 wk period.

d Refer to Fig. 1 .

Thus, we analyzed the trends of ADAMTS13 activity and VWF antigen and activity levels stratified by D-dimer. When stratified by D-dimer <2, 2–10, >10 μg/mL, ADAMTS13 activity levels incrementally decrease with higher D-dimer ( Supplementary Fig. S2G ). These D-dimer cut offs were based on our previous studies of D-dimer correlation with mortality. 18 Likewise, VWF:RCo and VWF antigen incrementally increase based on D-dimer levels ( Supplementary Fig. S2H and I ). In addition, similar trends are seen when VWF:CB and VWF:RCo were stratified by FM levels ( Supplementary Fig. S2K and L ).

### Increased High Molecular Weight Multimers in COVID-19 Inpatients

**Fig. 1:**
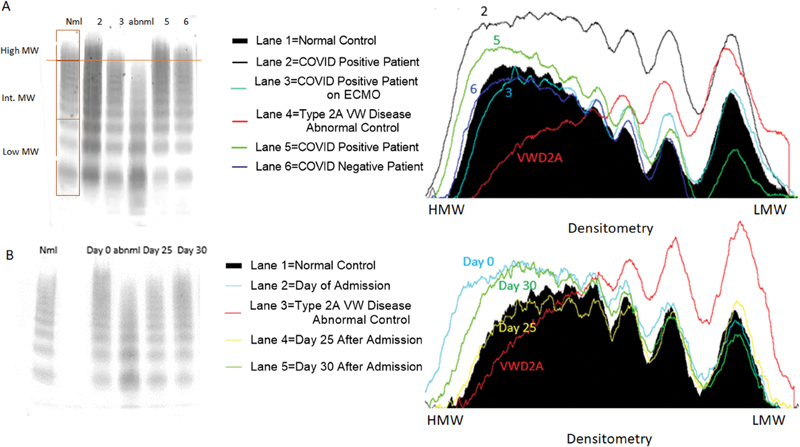
Cross sectional and longitudinal analysis of VWF multimers. For each multimer western blot, patient plasma was run on each lane, and the loading of all samples was normalized to measured VWF antigen levels. For each western blot, bands 1–3 were considered low molecular weight multimers, the bands between band 4 and the last band of the abnormal control were considered intermediate molecular weight multimers, and the bands above the last band of the abnormal control were considered HMW multimers. ( **A** ) The Western Blot to the left shows a pattern of VWF multimer cleavage in five patients. Lane 1 is the negative control, which was derived from normal pooled plasma, and lane 4 is the abnormal control, which was derived from the plasma of a patient with Type II von Willebrand disease. The abnormal control is missing HMW multimers. Lanes 2, 3, and 5 are from COVID-19 positive patients. The patient in lane 6 is from a COVID-19 negative patient with a normal multimer pattern. The COVID-19 positive patients have increased high molecular weight multimers, except for the patient in lane 3 who was on extracorporeal membrane oxygenation at the time of sample collection. The image to the right of the blot is the densitometry of the lanes represented in the western blot. The black filled in area represents the density of the normal control, and the red line indicates the abnormal control. The other lines indicate the densitometry of the multimers of the patients. ( **B** ) Longitudinal trends of coagulation parameters of a discharged alive COVID-19 patient. Western blot on the left shows the change in HMW multimer patterns throughout this patient's hospital stay. Lane 1 is the negative control and Lane 3 is the abnormal control. Multimer pattern between lane 1 and 2 was blocked as it was derived from an unrelated COVID-19 patient. Lane 2 corresponds to the day of admission, Lane 4 corresponds to day 25 after admission, and Lane 5 corresponds to day 30 after admission. The image to the right of the blot is the densitometry of the lanes represented in the western blot. The black filled in area represents the density of the normal control, and the red line indicates the abnormal control. The other lines indicate the densitometry of the multimers at various timepoints of the patient. Note that by day 25, HMW multimers decreased to normal size. COVID-19, coronavirus disease 2019; HMW, high molecular weight.

VWF multimer analysis was performed in the first 115 samples analyzed. We observed that many COVID-19 patients had an increased density of HMWMs compared with normal pooled plasma ( Table 2 , Fig. 1A ). Increased HMWM correlated with higher VWF:RCo ( *r* = 0.5; *p* < 0.0001, Supplementary Fig. S2F ) and increasing D-dimer ( *p* < 0.01, Supplementary Fig. S2J ). However, the relative increased HMWM was not significantly different between survivors and nonsurvivors ( Table 2 ). Therefore, no further VWF multimer analysis was performed in the remaining cases. Nonetheless, serial time points in a discharged alive patient showed how HMWM changed during hospitalization ( Fig. 1B ).

### Increased Schistocytes and LDH Are Associated with Low ADAMTS13 Activity and Higher Mortality

**Fig. 2:**
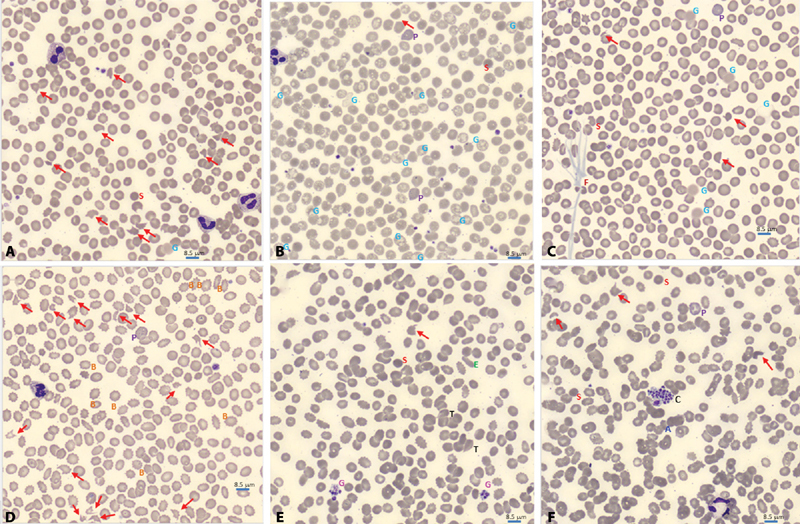
Blood smear abnormalities in COVID-19. Morphology evaluation of peripheral blood smear was performed by reviewing digital images from CellaVision. Schistocytes/RBC fragments ( *red arrow* ) was a predominant finding in many COVID-19 patients. Spherocytes and microspherocytes ( *red S* ) were very abundant, especially in smears with high number of schistocytes, thus only one is pointed in each smear for illustration ( **A–F** ). Ghost red blood cells ( *blue G* ) was also a common finding. Several morphologies of ghost cells were seen: smudge and diffuse hemoglobin staining without intact membrane ( **A and C** ), vacuolated red blood cells with intact membrane, and vacuolated ghost without intact membrane ( **B** ). Polychromatophils ( *purple P* ) were also seen ( **B, C, D, F** ). Fibrin strands ( *brown F* ) were seen in several patients ( **C** ). Echinocytes, aka Burr cells ( *orange B* ), were seen in association with renal injury ( **D** ). Giant platelets ( *pink G* ) were seen in many COVID-19 patients ( **E** ). Elliptocytes ( *green E* ) and tear drops ( *black T* ) were seen in several smears ( **E** ). Platelet clumps ( *black C* ) and agglutination ( *blue A* ) were also seen in some COVID-19 patients ( **F** ). Scale bar = 8.5 µm. COVID-19, coronavirus disease 2019.

Upon smear review, many RBC and platelet abnormalities were observed including fibrin strands, platelet clumps, giant platelets, echinocytes, elliptocytes, ghost cells, tear drops, schistocytes, RBC fragments, and RBC agglutination ( Fig. 2 ). Importantly, schistocytes and RBC fragments along with microspherocytes were among the most remarkable and predominant findings ( Fig. 2 ). Increased percentage of schistocytes/RBC fragments correlated with high VWF antigen and activity levels ( *r* = 0.24; *p* = 0.04) ( Supplementary Fig. S3A–C ). Increased percentage of schistocytes/RBC fragments also correlated with decreased platelet count ( *r* = −0.26; *p* = 0.02) and low ADAMTS13 activity ( *r* = −0.45; *p* < 0.0001) ( Supplementary Fig. S3D–F ). Increased percentage of schistocytes/RBC fragments correlated with markers of hemolysis, such as LDH ( *r* = 0.51; *p* < 0.0001) and indirect bilirubin ( Supplementary Fig. S3G–I ). Similarly, increased LDH strongly correlated with high VWF:RCo ( *r* = 0.25; *p* = 0.002) and VWF antigen ( *r* = 0.34; *p* < 0.0001) levels ( Supplementary Fig. S4A,B ). Importantly, LDH strongly correlated with indirect bilirubin ( *r* = 0.46; *p* < 0.0001), supporting their use as hemolysis markers ( Supplementary Fig. S4C ). High LDH correlated with increasing creatinine ( *r* = 0.16; *p* < 0.05), and creatinine also correlated inversely with hemoglobin ( *r* = −0.18; *p* = 0.02) and trended with decreasing platelet count ( *r* = −0.14; *p* = 0.06) ( Supplementary Fig. S4D–F ). The percentage of schistocytes/RBC fragments was higher in those who died than those who survived (1.06 [0.49, 2.63] vs. 0.56 [0.16, 1.12], *p* = 0.008) ( Table 2 ).

### Thrombosis, Coagulation Activation, and VWF

We documented thrombosis, type of thrombosis, anticoagulation, and temporal relationship of thrombosis to ADAMTS13 activity testing ( Supplementary Table S1 ). 19% (34 of 181) of the patients developed in vivo thrombosis during hospitalization. Patients with thrombosis exhibited significantly higher D-dimer (mean difference, 52.9; 95% confidence interval [CI] 27.3–l78.6 μg/mL), FM (517.4;168.8–865.9 μg/mL), VWF activity (62.3; 2.9–127.4%), higher number of cases with schistocytes/RBC fragments >1%, LDH (314.5;56.4–573.0 U/L), and creatinine (1.36;0.24–2.49 mg/dL) ( Supplementary Fig. S5 ).

A significant number of patients did not receive anticoagulation within 48 hours prior to clot detection (18 of 43 [42%]) ( Supplementary Table S1 ). Although the number of patients with documented thrombosis (both in vivo and ex vivo) was not significantly different based on ADAMTS13 activity levels, ex vivo clots, such as clots in the HD lines, were mainly observed in patients with ADAMTS13 activity levels lower than the normal range, which is less than 70% activity (10 patients [7.8%] vs. 1 [1.9%]; *p* = 0.181). Anticoagulation did not seem to change the risk of these thromboses ( Supplementary Table S1 ). However, VWF antigen and LDH levels were higher among the patients that received anticoagulation (mean difference, 105.9; 95% CI 13.4–198.4%, and 239.9; 54.0–425.8 U/L, respectively) ( Supplementary Fig. S6 ).

### Initial ADAMTS13 Activity Predicts Hospital Course and Discharge Outcome

**Fig. 3:**
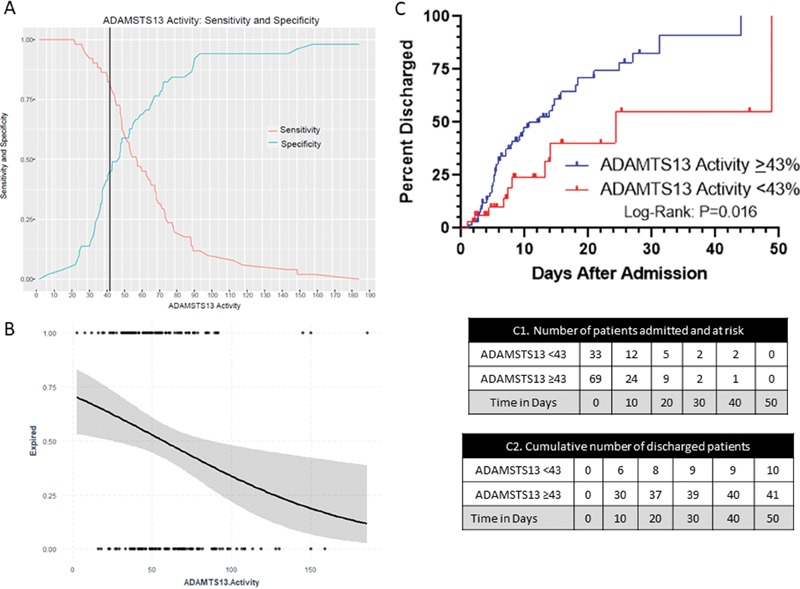
Initial ADAMTS13 activity as a predictor of mortality. ( **A** ) Youden index measuring the optimal cut point for ADAMTS13 activity as a differentiating marker when equal weight is given to sensitivity and specificity for the values in the cohort. The optimal cut-point for the initial ADAMTS13 activity within 72 hours since admission to predict mortality was found to be at 43% with an accuracy of 0.63, sensitivity of 0.82, and AUC of 0.63. ( **B** ) Logistic regression model of initial ADAMTS13 activity adjusted by age. Patients that deceased (each dot at the top classified as event 1) presented with lower ADAMTS13 activity levels compared to patients that were discharged alive (each dot at the bottom classified as event 0). The gray zone represents 95% of the confidence interval. ( **C** ) Kaplan-Meier curve shows cumulative number of discharged COVID-19 positive patients over time ( *n* = 102) based on initial ADAMTS13 activity. Patients with ADAMTS13 activity ≥ 43 show higher rate of discharge alive compared to patients with ADAMTS13 activity < 43 (log rank, *p* = 0.016). C1 table shows the number of COVID-19 positive patients admitted and at risk of mortality over time. C2 table shows the cumulative number of discharged alive patients in each group in increments of every 10 days. Each dot represents a discharged alive patient. ADAMTS13, a disintegrin and metalloproteinase with a thrombospondin type 1 motif, member 13; COVID-19, coronavirus disease 2019.

Given that ADAMTS13 activity levels seem to fluctuate during the course of hospitalization, we studied whether the initial ADAMTS13 activity within 72 hours of admission is predictive of mortality. A total of 102 patients had ADAMTS13 activity levels performed within 72 hours of admission ( Table 3 ). Using Youden's *J* statistic, we determined that the best cut-off of initial ADAMTS13 activity to predict mortality was 43%, *p* -value <0.01 ( Fig. 3A ). As expected, the demographic and clinical characteristics were similar to the larger original cohort ( Table 1 ). There was no difference by age or gender, although Hispanic patients represented 49% of patients with ADAMTS13 activity levels <43% ( Table 3 ). The logistic regression model of ADAMTS13 activity adjusted by age as a continuous variable showed that patients presenting with lower ADAMTS13 activity levels had higher risk of mortality ( Fig. 3B ). Only 30% (10/33) of patients with an ADAMTS13 activity <43% within 72 hours of admission survived compared with 60% of patients (41/69) with ADAMTS13 activity ≥43% who survived ( Fig. 3C ). Patients presenting with low ADAMTS13 activity (<43%) had significantly higher VWF:RCo activity (352.00 [225.00, 490.00] vs. 258.00 [200.00, 322.00]%; *p* = 0.04). The number of patients that required ventilation with an initial ADAMTS13 activity <43% was more than twice that of patients with initial ADAMTS13 activity ≥43% (12/33 [36%] vs. 11/69 [16%]; *p* = 0.04) ( Table 3 ).

**Table 3:** Clinical laboratory data within the first 72 hours of admission from patients with COVID-19 stratified by ADAMTS13 activity level

Characteristics		Low ADAMTS13 activity (<43%) ( *n* = 33 a ) (median [IQR] or n (%))	High ADAMTS13 activity (>43%) ( *n* = 69 a ) (median [IQR] or n (%))	*p* -Value
Age		71.0 [62.0, 80.0]	68.0 [59.0, 79.0]	0.31
Sex (Male)		22 (67)	41 (59)	0.50
Race	Black or African-American	7 (21)	28 (40)	0.09
	White	1 (3)	10 (15)	0.10
	Other/Patient declined	25 (76)	31 (45)	0.003
Ethnicity	Spanish/Hispanic/Latino	16 (49)	23 (33)	0.21
BMI (kg/m ^2^ )		30.9 [26.2, 32.8]	28.9 [26.9, 31.3]	0.25
Elixhauser comorbidity index		4.0 [2.0, 6.0]	4.0 [1.0, 6.0]	0.71
Mortality		23 (70)	28 (41)	0.01
Invasive ventilator use		12 (36)	11 (16)	0.04
In vivo thrombosis or ex vivo clot		5 (15)	11 (16)	>0.99
Vasopressor use		6 (18)	10 (15)	0.85
Hemodialysis or CRRT use		5 (15)	7 (10)	0.52
Anticoagulation use b		8 (24)	9 (13)	0.26
Steroid use c		10 (30)	19 (28)	0.96
**Clinical laboratory values measured at time of ADAMTS13 activity (units) [reference range]**				
ADAMTS13 activity (%) [70–110]		34.5 [26.8, 38.6]	66.2 [50.9, 79.9]	<0.001
Schistocyte count (%) [<0.5]		2.04 [1.05, 2.85] *n* = 13	0.48 [0.18, 0.68] *n* = 20	0.01
Schistocyte >1%		10 (30)	1 (1)	<0.001
Ghost cell count (%)		0.39 [0.2, 0.55] *n* = 13	0.27 [0.08, 2.77] *n* = 20	0.07
Lactate dehydrogenase (U/L) [140–280]		555.0 [417.0, 693.0]	452.0 [283.0, 625.5]	0.03
Indirect bilirubin (mg/dL) [0.2–0.8]		0.25 [0.10, 0.40]	0.25 [0.10, 0.40]	0.61
Hemoglobin (g/dL) [12–17.5]		12.9 [11.2, 14.6]	12.5 [10.4, 14.3]	0.35
Platelet count (k/µL) [150–450]		197.0 [149.0, 270.0]	211.0 [145.0, 304.0]	0.54
Platelet trajectory d	Decrease	16 (49)	9 (13)	<0.001
	Increase	11 (33)	37 (54)	0.06
Creatinine (mg/dL) [0.84–1.21]		1.6 [0.8, 2.4]	1.1 [0.9, 2.0]	0.40
Increased creatinine trajectory d		14 (42)	16 (23)	0.08
VWF activity (Ristocetin) (%) [50–150]		352.0 [225.0, 490.0]	258.0 [200.0, 322.0]	0.04
VWF antigen (%) [50–150]		442.0 [282.0, 656.0]	346.0 [256.0, 440.0]	0.06
D-dimer (µg/mL) [<0.27]		2.6 [1.8, 13.9]	1.9 [0.8, 6.7]	0.09
Fibrin monomer (µg/mL) [<10]		5.0 [5.0, 61.8]	5.0 [5.0, 24.5]	0.29
Factor VIII activity (%) [50–150]		144.0 [112.0, 153.0] *n* = 11	154.0 [98.0, 188.0] *n* = 37	0.81
HMW multimer fold change from normal e		0.90 [0.68, 1.70] *n* = 13	1.01 [0.87, 1.20] *n* = 36	0.63
Fibrinogen (mg/dL) [200–400]		510.0 [377.5, 681.0] *n* = 19	588.0 [458.8, 747.5] *n* = 48	0.43
**Initial clinical laboratory values measured upon admission (units) [reference range]**				
eGFR (mL/min/1.73 m ^2^ ) [90–120]		39.0 [26.0, 83.0]	56.0 [30.8, 83.4]	0.73
Aspartate Transaminase (IU/L) [8–48]		53.5 [42.5, 106.8]	44.0 [28., 69.0]	0.06
Mean platelet volume (fL) [7–12]		11.2 [10.7, 11.6]	10.7 [10.0, 11.8]	0.15
Lymphocyte (count) [1.5–4.5]		0.8 [0.6, 0.9]	1.1 [0.7, 1.5]	0.02
International normalized ratio [<1.1]		1.1 [1.0, 1.3]	1.2 [1.1, 1.3]	0.38
C-reactive protein (mg/L) [<10]		17.0 [5.3, 27.1]	13.7 [4.5, 20.4]	0.19
Troponin (ng/mL) [<0.04]		0.04 [0.01, 0.08]	0.01 [0.01, 0.04]	0.05
Pulse oximeter (%) [95–100]		92.0 [84.0, 96.0]	96.0 [91.0, 99.0]	0.03
Diastolic blood pressure (mm Hg) [60–80]		70.0 [46.0, 80.0]	72.0 [61.0, 82.0]	0.45

Abbreviations: ADAMTS13, a disintegrin and metalloproteinase with a thrombospondin type 1 motif, member 13; BMI, body mass index; CRRT, continuous renal replacement therapies; eGFR, estimated glomerular filtration rate; HMW, high molecular weight; IQR, interquartile range; VWF, Von Willebrand factor.

a Unless otherwise stated.

b Within 48 h prior to clot or ADAMTS13 activity measurement.

c Within 24 h prior to ADAMTS13 activity measurement.

d Based on a change >10% over 1 wk period.

e Refer to Fig. 1 .

Severe thrombocytopenia at presentation was rare, with only one patient having a platelet count of 1 k/µL and an ADAMTS13 activity level of 118%. Although admission platelet count was not significantly different between patients with ADAMTS13 activity <43% versus ≥43%, the trajectory (defined as a change >10% within 7 days) was significantly different. Sixteen (49%) patients admitted with ADAMTS13 activity <43% had a decrease in their platelets compared with nine (13%) patients with ADAMTS13 activity ≥43%; *p* < 0.001 ( Table 3 ). The majority (16/25, 64%) of patients with a negative platelet trajectory died.

D-dimer, FM, fibrinogen, and FVIII activity were not significantly different in patients with an initial ADAMTS13 activity <43% compared with patients with an initial ADAMTS13 activity ≥43%. Although a strong correlation was observed between initial D-dimer and FM, no correlation was observed between initial D-dimer and prothrombin time (PT), FVIII activity, platelet count, and hemoglobin ( Supplementary Fig. S7 ).

### ADAMTS13 Activity Levels <30% Are Not Caused by Immune-Mediated Antibodies

To investigate the etiology of the decreased ADAMTS13 activity, we assessed inhibitor status for the protease in all cases with an ADAMTS13 activity <30%, which is a routine cut off for further work-up for antibody detection. 12% (22 of 181) of patients in our cohort had ADAMTS13 activity levels <30% and most had mild <40% inhibition ( Table 4 ), but when these samples were tested for specific antibodies against ADAMTS13 by ELISA, none were found in any of these patients. Since IL-6 can inhibit ADAMTS13 activity, we correlated ADAMTS13 activity with IL-6 levels 19 ; however, we did not observe a linear correlation between IL-6 and degree of ADAMTS13 activity inhibition ( *r* = 0.06). Likewise, ADAMTS13 activity level did not directly correlate with eGFR or AST (not shown). However, more than 80% of these patients had albumin levels below the normal reference range. Assessment for dysfunctional ADAMTS13 was unrevealing: ADAMTS13 antigen levels were correspondingly low in the nine patients in whom it was measured (0.1–0.4, normal range 0.6–1.6 UI/mL, data not shown).

**Table 4:** ADAMTS13 activity inhibitor, kidney function, liver function, and immunological analysis of patients with very low (≤30) ADAMTS13 activity

Patient	Dispo.	ADAMTS-13 activity (%) [70–110]	% Inhibition ADAMTS-13 activity [<30%] a	ADAMTS-13 antibody	Clotting evidence	Schistocyte count (%) [<0.5]	Platelet (count) [150–450]	eGFR (mL/min/1.73 m ^2^ ) [90–120]	Albumin (g/dL) [3.4–5.4]	VWF antigen (%) [50–150]	D-dimer (µg/mL) [<0.27]	IL-6 (pg/mg) [<17] b
**Patient A**	Died	2.6	36.2	Neg	No	<1	161.0	42.0	3.9	513.0	2.0	77.3 (3)
**Patient B**	Died	7.7	37.2	Neg	No	<1	218.0	>120	4.3	282.0	20.0	71.4 (−2)
**Patient C**	Died	11.9	37.1	Neg	No	<1	102.0	74.0	2.9	1020.0	0.6	
**Patient D**	Alive	16.0	29.1	Neg	No	4.3	49.0	5.0	2.7	386.0	0.7	43.6 (0)
**Patient E**	Died	16.2	33.6	Neg	No	<1	267.0	43.0	3.5	442.0	105.0	12.1 (−2)
**Patient F**	Alive	18.0	QNS	Neg	No	1.3	238.0	76.0	2.8	648.0	16.2	47.9 (−6)
**Patient G**	Died	18.6	21.9	Neg	No	6.9	60.0	41.0	2.1	1325.0	64.6	7982.5 (−1)
**Patient H**	Died	19.7	7.0	Neg	Ex vivo clot	1.5	485.0	16.0	2.6	498.0	7.9	226.0 (2)
**Patient I**	Alive	22.6	4.7	Neg	Pulmonary embolism	<1	264.0	114.0	2.9	309.0	2.1	27.1 (0)
**Patient J**	Died	23.0	27.9	Neg	No	<1	190.0	64.0	2.5	244.0	3.7	132.3 (−17)
**Patient K**	Died	23.2	21.6	Neg	No	8.6	37.0	26.0	2.6	273.0	0.8	120.6 (−1)
**Patient L**	Alive	23.4	40.5	Neg	No	<1	452.0	101.0	2.7	264.0	5.4	
**Patient M**	Died	24.3	25.7	Neg	No	<1	124.0	84.0	3.1	656.0	1.0	187.9 (0)
**Patient N**	Died	25.4	32.1	Neg	Stroke	2.9	187.0	62.0	2.5	880.0	189.0	
**Patient O**	Died	25.6	23.1	Neg	No	<1	197.0	>120	2.8	598.0	197.6	1338.5 (0)
**Patient P**	Died	26.1	37.0	Neg	Arterial thrombus	<1	161.0	10.0	3.1	120.0	3.5	16.90 (0)
**Patient Q**	Alive	26.8	18.2	Neg	No	<1	82.0	44.0	3.4	78.0	0.8	25.50 (0)
**Patient R**	Died	27.6	29.0	Neg	No	2.9	498.0	14.0	2.5	514.0	20.0	283.4 (−1)
**Patient S**	Alive	27.7	33.0	Neg	No	<1	190.0	>120	3.3	262.0	0.5	
**Patient T**	Died	28.8	27.3	Neg	No	<1	187.0	18.0	2.0	456.0	3.5	1826 (−12)
**Patient U**	Alive	29.2	22.3	Neg	No	1.6	147.0	10.0	3.1	255.0	0.7	45.7 (0)
**Patient V**	Died	30.8	11.8	Neg	Ex vivo clot	6.8	96.0	75.0	2.1	738.0	20.0	173.7 (−32)

Abbreviations: ADAMTS13, a disintegrin and metalloproteinase with a thrombospondin type 1 motif, member 13; LDH, lactate dehydrogenase; Hgb, hemoglobin; eGFR, estimated glomerular filtration rate; AST, aspartate aminotransferase; VWF, Von Willebrand factor; IL-6, Interleukin 6; QNS, quantity not sufficient.

a Missing values are due to insufficient quantity of sample.

b Missing values are due to the test not being ordered for the patient. Number in parenthesis indicates the days relative to ADAMTS13 activity measurement.

## Discussion

The main hypothesis of this retrospective study is that VWF biomarkers are associated with coagulation in COVID-19. We performed a balanced retrospective study of COVID-19 hospitalized patients with similar demographics and comorbidities and a wide range of D-dimer levels to study how VWF biomarkers correlate with coagulation, intravascular hemolysis, and outcome. Indeed, we show a clear association of elevated VWF antigen and activity levels with high D-dimer and FM levels. We also show that mild ADAMTS13 activity deficiency is common in COVID-19 inpatients.

Elevated VWF antigen and activity levels have been documented in COVID-19. 9 11 20 Likewise, inflammatory markers such as CRP and IL-6 are known to be elevated in COVID-19. Thus, a potential explanation for elevated VWF antigen and activity levels in COVID-19 could be that this represents an acute phase response. 21 22 However, the magnitude of increases in D-dimer, FM levels, and VWF antigen and activity cannot be explained solely by acute phase response and/or inflammation. In addition, ADAMTS13 activity is not expected to significantly decrease in acute inflammation, yet the majority of COVID-19 patients had decreased ADAMTS13 activity, indicating a profound endothelial dysregulation or an intrinsic ADAMTS13 activity deficiency. Possible mechanisms of ADAMTS13 activity deficiency include decreased production, inhibition, or consumption of ADAMTS13. 12% of patients in our cohort had ADAMTS13 activity levels less than 30% but none had detectable anti-ADAMTS13 antibodies. Many of these patients had increased IL-6 levels, but the IL-6 level did not correlate linearly with reduced ADAMTS13 activity, thus favoring consumption or decreased production rather than inhibition. ADAMTS13 antigen levels were also reduced and >80% of patients with ADAMTS13 activity levels <30% had albumin levels below normal reference range, thus liver dysfunction may explain a low ADAMTS13 activity in these patients. In addition, consumption of ADAMTS13 due to excess of its substrate, VWF, or excess of plasmin, has been observed in sepsis, disseminated intravascular coagulopathies (DICs), and thrombotic microangiopathy (TMA). 23 24 25 26 27 Indeed, elevated VWF antigen and activity levels, D-dimer levels, FM levels along with moderately reduced ADAMTS13 activity levels is a repertoire of hallmarks shared by critical illnesses that result in severe microvascular endothelial cell injuries. 28 29 30

Thrombocytopenia is not common in COVID-19 and was not directly associated with low ADAMTS13 activity levels in our cohort. 31 Also, lack of severe ADAMTS13 activity deficiency (only two patients had ADAMTS13 activity <10%) and lack of anti-ADAMTS13 antibodies in our patients excludes thrombotic thrombocytopenic purpura (TTP) 32 and may be more suggestive of secondary TMA, sepsis, or DIC.

TMA is defined by the triad of microangiopathic anemia, thrombocytopenia, and end-organ damage. 33 In our cohort we found evidence of microangiopathic anemia (schistocytes/RBC fragments) and intravascular hemolysis (high LDH, indirect bilirubin) in the majority of patients with low ADAMTS13 activity. The correlation of schistocytes/RBC fragments with markers of hemolysis (LDH, indirect bilirubin), and elevated VWF antigen and activity levels with a concomitant presence of decreased ADAMTS13 activity may indicate a TMA pattern. Also, the correlation of high D-dimer and FM levels with LDH and the occasional finding of fibrin strands in peripheral blood smears suggests that high D-dimer levels may be a direct product of small vessel thrombosis (arterial and venous), which have been documented in COVID-19 autopsies. 34 35 36 37 38 39 Microvascular thrombosis leads to ischemic end-organ damage, most commonly affecting kidneys, but other organs can also be affected. COVID-19 primarily manifests as respiratory failure, however, renal and cardiovascular complications are common in COVID-19. In our cohort approximately 40% of patients required ventilation, 20% developed thrombosis, and 15% required hemodialysis, of which 28% developed ex vivo clots. We observed a trend of both in vivo and ex vivo thrombosis in cases with lower ADAMTS13 activity but did not reach a significance of *p* < 0.05, probably due to the small sample size. The lack of thrombocytopenia in the majority of the patients with low ADAMTS13 activity argues against TMA. Although thrombocytopenia was not common in our cohort, evidence of platelet consumption in peripheral blood smears (large immature platelets and platelet clumps) and decreasing platelet trajectories was prevalent among patients with low ADAMTS13 activity.

High D-dimer levels, coagulation factor consumption, platelet consumption, and anemia along with multiple organ damage are hallmarks of DIC. 40 However, in our cohort high D-dimer levels did not correlate with prolonged PT, and unlike overt DIC, COVID-19 patients presented with elevated fibrinogen, FVIII activity, and FM along with D-dimer ( Supplementary Fig. S7 ). Furthermore, high D-dimer did not correlate with decreasing hemoglobin or platelets, excluding the classic overt DIC. Nonetheless, nonovert DIC which has been described in COVID-19, may explain these findings. 41

Approximately 60% of patients that developed thrombosis were on anticoagulation at least 48 hours prior to clot detection. However, patients receiving anticoagulation had higher LDH and VWF antigen and activity levels, suggesting these patients were sicker ( Supplementary Fig. S6 ). Many other studies have shown that despite anticoagulation, certain COVID-19 patients still thrombose. 42 Anticoagulation alone is not an effective treatment for DIC. 44 Nafamostat, a synthetic serine protease inhibitor, used to treat DIC and pancreatitis, has been shown as beneficial in COVID-19 treatment in several case reports. 45 Additional treatment may be required to decrease high levels of VWF antigen and activity and increase ADAMTS13 activity. A nanobody, caplacizumab, that inhibits the binding of VWF to gp-1b on platelets has been effective in treating TMAs. 46 ADAMTS13 replacement via plasma exchange is a standard TTP treatment. 47 Although the main rationale of convalescent plasma (CP) treatment is to provide passive immunity to acutely ill COVID-19 patients, replacement of ADAMTS13, and other plasma proteins can possibly contribute to benefits attributed to CP. 48 49 50 Although we did not measure complement levels in our cohort, as serum samples were not preserved, we noticed ghost cells in several cases, which suggest complement activation of RBCs. 51 Eculizumab, a monoclonal antibody, binds C5, inhibiting the terminal complement complex, and has been shown to be effective in treating COVID-19 in several case reports. 52 53 54

An advantage of our study is that cases were selected based on a repository of frozen plasma, and thus multiple tests with serial dilutions were performed, allowing us to accurately correlate D-dimer concentration, FM concentration, VWF activity, VWF antigen, VWF multimers, FVIII activity, and ADAMTS13 activity levels, all derived from the same samples. In addition, serial dilutions of samples that reach the upper limit of detection allow us to accurately measure the actual elevated levels of D-dimer, FM, and VWF antigen and activity. Herein, we showed cases with unprecedented levels of VWF antigen and activity >1,000%, FM >2,000 μg/mL, and D-dimer >300 μg/mL FEU.

**Fig. 4:**
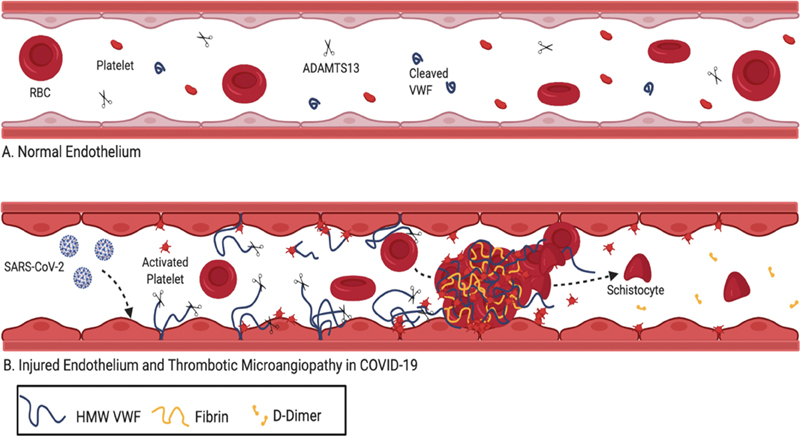
Unique COVID-19 microangiopathy. ( **A** ) Normal endothelium. ( **B** ) SARS-CoV-2 enters the endothelial cells of capillaries via the ACE2R. Injured endothelial cells release HMW multimers of VWF which unfold in shear forces of the microvasculature. HMW multimers recruit platelets to the wounded endothelium. Unfolded HMW multimers consume circulating ADAMTS13 activity, allowing for increased platelet binding to uncleaved HMW multimers downstream. In turn, activated platelets aggregate activating coagulation and forming microvascular thrombi. In high shear stress, schistocytes are formed as a result of RBC shearing while forced through small vessels with thrombi. High D-dimers result from plasmin degradation of microthrombi. ACE2R, ACE2 receptor; ADAMTS13, a disintegrin and metalloproteinase with a thrombospondin type 1 motif, member 13; COVID-19, coronavirus disease 2019; SARS-CoV-2, severe acute respiratory syndrome coronavirus 2. HMW, high molecular weight; VWF, von Willebrand factor.

In summary, we present the most comprehensive and largest study to date analyzing correlations of D-dimer levels with VWF activity and antigen, size of VWF multimers, ADAMTS13 activity levels, markers of intravascular hemolysis, and smear pathology in hospitalized COVID-19 patients. A subset of COVID-19 inpatients presents a unique microangiopathy characterized by elevated VWF antigen and activity, D-dimer, schistocytes/RBC fragments, and evidence of macrothrombosis but also microthrombosis ( Fig. 4 ). In particular, the markedly elevated D-dimer levels, along with mildly reduced ADAMTS13 activity and lack of thrombocytopenia argues against TTP or TMA. In contrast to TTP, D-dimer levels are significantly higher in DIC. 28 Furthermore, presence of elevated D-dimer (>4 mg/mL) is a negative predictor of TTP in the Bentley score, the first clinical diagnostic score system for TTP. 28 The elevated VWF antigen and activity levels are derived from endothelial cells that are activated and/or damaged by the SARS-CoV2 virus infection ( Fig. 4 ). The resulting moderate decrease in ADAMTS13 activity is likely a combination of decreased production due to liver impairment and/or consumption by excess of VWF ( Fig. 4 ). Presence of schistocytes/RBC fragments, elevated D-dimer, and hallmarks of platelet activation and consumption correlate with a growing collective evidence of platelet-fibrin macro- and microthrombosis in the lungs and other organs of COVID-19 patients. 28 39 In our cohort we demonstrate that low ADAMTS13 activity and increased schistocytes/RBC fragments on admission correlated with mortality. Thus, in addition to elevated D-dimer, presence of schistocytes/RBC fragments on admission may warrant further work-up including ADAMTS13 activity and VWF antigen and activity levels since these patients may be at increased risk of mortality and may benefit from more aggressive therapy.

Like any other retrospective studies, limitations include intrinsic confounders and bias. Choosing samples from a limited repository bank could create bias. We tried to compensate by randomly selecting a balanced cohort with equal distribution of survivors and nonsurvivors and similar demographics. We could only demonstrate correlations but no causality. Major confounders include: a wide spectrum of disease severity at presentation, and possibly over imposed sepsis. Thus, we cannot exclude the possibility that low ADAMTS13 activity is a simple passive biomarker and an indirect consequence of disease severity. Therefore, prospective randomized clinical studies are needed to determine the relationship and causality between ADAMTS13 activity, complement, endothelial, and coagulation activation and to study the efficacy of treatments aiming at preventing and/or ameliorating COVID-19 microangiopathy.

## References

[1] Chen N Zhou M Dong X Epidemiological and clinical characteristics of 99 cases of 2019 novel coronavirus pneumonia in Wuhan, China: a descriptive study Lancet 2020 395(10223):507 513 32007143 10.1016/S0140-6736(20)30211-7 PMC7135076

[2] Huang C Wang Y Li X Clinical features of patients infected with 2019 novel coronavirus in Wuhan, China Lancet 2020 395(10223):497 506 31986264 10.1016/S0140-6736(20)30183-5 PMC7159299

[3] Labò N Ohnuki H Tosato G Vasculopathy and coagulopathy associated with SARS-CoV-2 infection Cells 2020 9 07 E1583 32629875 10.3390/cells9071583 PMC7408139

[4] Buja L M Wolf D A Zhao B The emerging spectrum of cardiopulmonary pathology of the coronavirus disease 2019 (COVID-19): report of 3 autopsies from Houston, Texas, and review of autopsy findings from other United States cities Cardiovasc Pathol 2020 48 107233 32434133 10.1016/j.carpath.2020.107233 PMC7204762

[5] Deshpande C Thromboembolic findings in COVID-19 autopsies: pulmonary thrombosis or embolism? Ann Intern Med 2020 173 05 394 395 32422061 10.7326/M20-3255 PMC7249508

[6] Global COVID-19 Thrombosis Collaborative Group, Endorsed by the ISTH, NATF, ESVM, and the IUA, Supported by the ESC Working Group on Pulmonary Circulation and Right Ventricular Function Bikdeli B Madhavan M V Jimenez D COVID-19 and thrombotic or thromboembolic disease: implications for prevention, antithrombotic therapy, and follow-up: JACC state-of-the-art review J Am Coll Cardiol 2020 75 23 2950 2973 32311448 10.1016/j.jacc.2020.04.031 PMC7164881

[7] Zhang L Yan X Fan Q D-dimer levels on admission to predict in-hospital mortality in patients with Covid-19 J Thromb Haemost 2020 18 06 1324 1329 32306492 10.1111/jth.14859 PMC7264730

[8] Zhou F Yu T Du R Clinical course and risk factors for mortality of adult inpatients with COVID-19 in Wuhan, China: a retrospective cohort study Lancet 2020 395(10229):1054 1062 32171076 10.1016/S0140-6736(20)30566-3 PMC7270627

[9] Goshua G Pine A B Meizlish M L Endotheliopathy in COVID-19-associated coagulopathy: evidence from a single-centre, cross-sectional study Lancet Haematol 2020 7 08 e575 e582 32619411 10.1016/S2352-3026(20)30216-7 PMC7326446

[10] Sadler J E von Willebrand factor: two sides of a coin J Thromb Haemost 2005 3 08 1702 1709 16102036 10.1111/j.1538-7836.2005.01369.x

[11] Escher R Breakey N Lämmle B ADAMTS13 activity , von Willebrand factor, factor VIII and D-dimers in COVID-19 inpatients Thromb Res 2020 192 174 175 32505009 10.1016/j.thromres.2020.05.032 PMC7245313

[12] Martinelli N Montagnana M Pizzolo F A relative ADAMTS13 activity deficiency supports the presence of a secondary microangiopathy in COVID 19 Thromb Res 2020 193 170 172 32707276 10.1016/j.thromres.2020.07.034 PMC7367811

[13] Huisman A Beun R Sikma M Westerink J Kusadasi N Involvement of ADAMTS13 activity and von Willebrand factor in thromboembolic events in patients infected with SARS-CoV-2 Int J Lab Hematol 2020 42 05 e211 e212 32441844 10.1111/ijlh.13244 PMC7280565

[14] Miller C H Platt S J Daniele C Kaczor D Evaluation of two automated methods for measurement of the ristocetin cofactor activity of von Willebrand factor Thromb Haemost 2002 88 01 56 59 12152679

[15] Lawrie A S Hoser M J Savidge G F Phast assessment of vWf:Ag multimeric distribution Thromb Res 1990 59 02 369 373 2237815 10.1016/0049-3848(90)90139-4

[16] Hantaweepant C Sasijareonrat N Chutvanichkul B Karaketklang K Chinthammitr Y Comparison between optical microscopy and the Sysmex XN-3000 for schistocyte determination in patients suspected of having schistocytosis Health Sci Rep 2019 3 01 e138 32166186 10.1002/hsr2.138 PMC7060895

[17] Favaloro E J Bonar R Chapman K Meiring M Funk Adcock D Differential sensitivity of von Willebrand factor (VWF) ‘activity’ assays to large and small VWF molecular weight forms: a cross-laboratory study comparing ristocetin cofactor, collagen-binding and mAb-based assays J Thromb Haemost 2012 10 06 1043 1054 22487084 10.1111/j.1538-7836.2012.04729.x

[18] Billett H H Reyes-Gil M Szymanski J Anticoagulation in COVID-19: effect of enoxaparin, heparin, and apixaban on mortality Thromb Haemost 2020 120 12 1691 1699 33186991 10.1055/s-0040-1720978 PMC7869055

[19] Bernardo A Ball C Nolasco L Moake J F Dong J F Effects of inflammatory cytokines on the release and cleavage of the endothelial cell-derived ultralarge von Willebrand factor multimers under flow Blood 2004 104 01 100 106 15026315 10.1182/blood-2004-01-0107

[20] Ladikou E E Sivaloganathan H Milne K M Von Willebrand factor (vWF): marker of endothelial damage and thrombotic risk in COVID-19? Clin Med (Lond) 2020 20 05 e178 e182 32694169 10.7861/clinmed.2020-0346 PMC7539718

[21] Liu F Li L Xu M Prognostic value of interleukin-6, C-reactive protein, and procalcitonin in patients with COVID-19 J Clin Virol 2020 127 104370 32344321 10.1016/j.jcv.2020.104370 PMC7194648

[22] Zhang J Yu M Tong S Liu L Y Tang L V Predictive factors for disease progression in hospitalized patients with coronavirus disease 2019 in Wuhan, China J Clin Virol 2020 127 104392 32361327 10.1016/j.jcv.2020.104392 PMC7187844

[23] Farkas P Csuka D Mikes B Complement activation, inflammation and relative ADAMTS13 activity deficiency in secondary thrombotic microangiopathies Immunobiology 2017 222 02 119 127 27771173 10.1016/j.imbio.2016.10.014

[24] Kremer Hovinga J A Zeerleder S Kessler P ADAMTS-13, von Willebrand factor and related parameters in severe sepsis and septic shock J Thromb Haemost 2007 5 11 2284 2290 17764538 10.1111/j.1538-7836.2007.02743.x

[25] Feys H B Vandeputte N Palla R Inactivation of ADAMTS13 activity by plasmin as a potential cause of thrombotic thrombocytopenic purpura J Thromb Haemost 2010 8 09 2053 2062 20553378 10.1111/j.1538-7836.2010.03942.x

[26] Ono T Mimuro J Madoiwa S Severe secondary deficiency of von Willebrand factor-cleaving protease (ADAMTS13 activity) in patients with sepsis-induced disseminated intravascular coagulation: its correlation with development of renal failure Blood 2006 107 02 528 534 16189276 10.1182/blood-2005-03-1087

[27] Habe K Wada H Ito-Habe N Plasma ADAMTS13 activity, von Willebrand factor (VWF) and VWF propeptide profiles in patients with DIC and related diseases Thromb Res 2012 129 05 598 602 22070827 10.1016/j.thromres.2011.10.011

[28] Wada H Mori Y Shimura M Poor outcome in disseminated intravascular coagulation or thrombotic thrombocytopenic purpura patients with severe vascular endothelial cell injuries Am J Hematol 1998 58 03 189 194 9662269 10.1002/(sici)1096-8652(199807)58:3<189::aid-ajh5>3.0.co;2-n

[29] Smeets N JL Fijnheer R Sebastian S De Mast Q Secondary thrombotic microangiopathy with severely reduced ADAMTS13 activity in a patient with Capnocytophaga canimorsus sepsis: a case report Transfusion 2018 58 10 2426 2429 30222856 10.1111/trf.14829

[30] Schwameis M Schörgenhofer C Assinger A Steiner M M Jilma B VWF excess and ADAMTS13 activity deficiency: a unifying pathomechanism linking inflammation to thrombosis in DIC, malaria, and TTP Thromb Haemost 2015 113 04 708 718 25503977 10.1160/TH14-09-0731 PMC4745134

[31] Iba T Levy J H Levi M Thachil J Coagulopathy in COVID-19 J Thromb Haemost 2020 18 09 2103 2109 32558075 10.1111/jth.14975 PMC7323352

[32] Joly B S Coppo P Veyradier A Thrombotic thrombocytopenic purpura Blood 2017 129 21 2836 2846 28416507 10.1182/blood-2016-10-709857

[33] Lopes da Silva R Viral-associated thrombotic microangiopathies Hematol Oncol Stem Cell Ther 2011 4 02 51 59 21727765 10.5144/1658-3876.2011.51

[34] Ackermann M Verleden S E Kuehnel M Pulmonary vascular endothelialitis, thrombosis, and angiogenesis in Covid-19 N Engl J Med 2020 383 02 120 128 32437596 10.1056/NEJMoa2015432 PMC7412750

[35] do Espírito Santo D A Lemos A CB Miranda C H In vivo demonstration of microvascular thrombosis in severe COVID-19 J Thromb Thrombolysis 2020 50 04 790 794 32789730 10.1007/s11239-020-02245-x PMC7424241

[36] Fox S E Akmatbekov A Harbert J L Li G Quincy Brown J Vander Heide R S Pulmonary and cardiac pathology in African American patients with COVID-19: an autopsy series from New Orleans Lancet Respir Med 2020 8 07 681 686 32473124 10.1016/S2213-2600(20)30243-5 PMC7255143

[37] Grosse C Grosse A Salzer H JF Dünser M W Motz R Langer R Analysis of cardiopulmonary findings in COVID-19 fatalities: high incidence of pulmonary artery thrombi and acute suppurative bronchopneumonia Cardiovasc Pathol 2020 49 107263 32784110 10.1016/j.carpath.2020.107263 PMC7365076

[38] Mackman N Antoniak S Wolberg A S Kasthuri R Key N S Coagulation abnormalities and thrombosis in patients infected with SARS-CoV-2 and other pandemic viruses Arterioscler Thromb Vasc Biol 2020 40 09 2033 2044 32657623 10.1161/ATVBAHA.120.314514 PMC7447001

[39] Rapkiewicz A V Mai X Carsons S E Megakaryocytes and platelet-fibrin thrombi characterize multi-organ thrombosis at autopsy in COVID-19: a case series EClinicalMedicine 2020 24 100434 32766543 10.1016/j.eclinm.2020.100434 PMC7316051

[40] Schutte T Thijs A Smulders Y M Never ignore extremely elevated D-dimer levels: they are specific for serious illness Neth J Med 2016 74 10 443 448 27966438

[41] Mazzaccaro D Giacomazzi F Giannetta M Non-overt coagulopathy in non-ICU patients with mild to moderate COVID-19 pneumonia J Clin Med 2020 9 06 E1781 32521707 10.3390/jcm9061781 PMC7355651

[42] Al-Ani F Chehade S Lazo-Langner A Thrombosis risk associated with COVID-19 infection. A scoping review Thromb Res 2020 192 152 160 32485418 10.1016/j.thromres.2020.05.039 PMC7255332

[43] Merrill J T Erkan D Winakur J James J A Emerging evidence of a COVID-19 thrombotic syndrome has treatment implications Nat Rev Rheumatol 2020 16 10 581 589 32733003 10.1038/s41584-020-0474-5 PMC7391481

[44] Yatabe T Inoue S Sakamoto S The anticoagulant treatment for sepsis induced disseminated intravascular coagulation; network meta-analysis Thromb Res 2018 171 136 142 30312798 10.1016/j.thromres.2018.10.007

[45] Okajima M Takahashi Y Kaji T Ogawa N Mouri H Nafamostat mesylate-induced hyperkalemia in critically ill patients with COVID-19: four case reports World J Clin Cases 2020 8 21 5320 5325 33269265 10.12998/wjcc.v8.i21.5320 PMC7674713

[46] HERCULES Investigators Scully M Cataland S R Peyvandi F Caplacizumab treatment for acquired thrombotic thrombocytopenic purpura N Engl J Med 2019 380 04 335 346 30625070 10.1056/NEJMoa1806311

[47] Furlan M Robles R Morselli B Sandoz P Lämmle B Recovery and half-life of von Willebrand factor-cleaving protease after plasma therapy in patients with thrombotic thrombocytopenic purpura Thromb Haemost 1999 81 01 8 13 10348715

[48] Fischer J C Zänker K van Griensven M The role of passive immunization in the age of SARS-CoV-2: an update Eur J Med Res 2020 25 01 16 32404189 10.1186/s40001-020-00414-5 PMC7220618

[49] Tanne J H Covid-19: FDA approves use of convalescent plasma to treat critically ill patients BMJ 2020 368 m1256 32217555 10.1136/bmj.m1256

[50] Valk S J Piechotta V Chai K L Convalescent plasma or hyperimmune immunoglobulin for people with COVID-19: a rapid review Cochrane Database Syst Rev 2020 5 CD013600 32406927 10.1002/14651858.CD013600 PMC7271896

[51] Himmelfarb J McMonagle E Holbrook D Hakim R Increased susceptibility to erythrocyte C5b-9 deposition and complement-mediated lysis in chronic renal failure Kidney Int 1999 55 02 659 666 9987090 10.1046/j.1523-1755.1999.00277.x

[52] Diurno F Numis F G Porta G Eculizumab treatment in patients with COVID-19: preliminary results from real life ASL Napoli 2 Nord experience Eur Rev Med Pharmacol Sci 2020 24 07 4040 4047 32329881 10.26355/eurrev_202004_20875

[53] Giudice V Pagliano P Vatrella A Combination of ruxolitinib and eculizumab for treatment of severe SARS-CoV-2-related acute respiratory distress syndrome: a controlled study Front Pharmacol 2020 11 857 32581810 10.3389/fphar.2020.00857 PMC7291857

[54] Laurence J Mulvey J J Seshadri M Anti-complement C5 therapy with eculizumab in three cases of critical COVID-19 Clin Immunol 2020 219 108555 32771488 10.1016/j.clim.2020.108555 PMC7410014

