# Supplementary material for: Low ADAMTS13 Activity Correlates with Increased Mortality in COVID-19 Patients
Source: TH Open. 2021 Mar 9;5(1):e89–e103. doi: 10.1055/s-0041-1723784 (PMC7943318; doi:10.1055/s-0041-1723784)
Supplement: Supplementary file 1 — Supplementary Material [file 10-1055-s-0041-1723784-s200092.pdf]

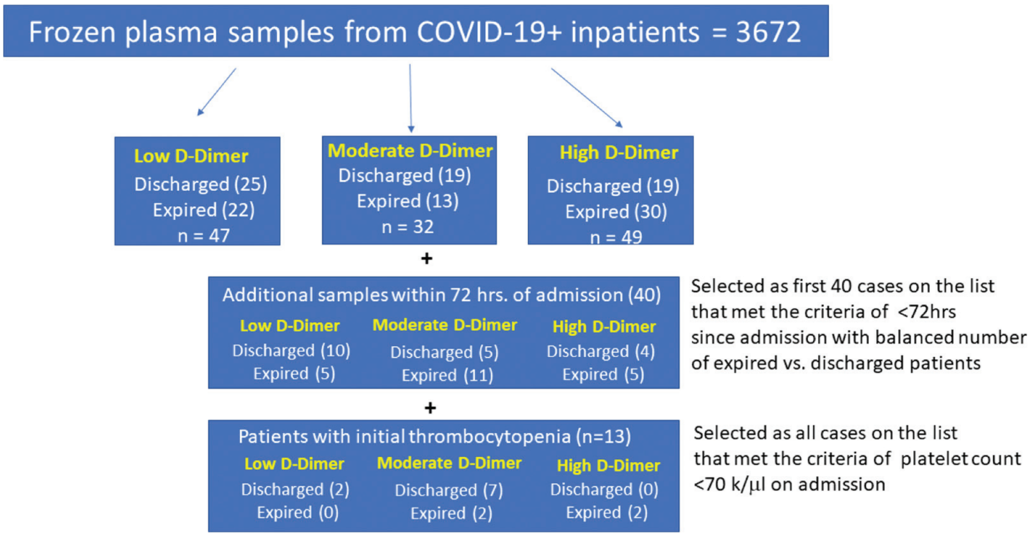

Supplementary Fig. S1 Flowchart of sample selection. 3,672 plasma samples were frozen, deidentified, coded with a unique arbitrary number, sorted by this arbitrary number (lowest to highest) and grouped into two categories: deceased versus discharged alive cases. To study the association of disease severity with VWF antigen and activity, and ADAMTS13 activity, samples were then selected down the list (top to bottom) with balanced number of discharged alive versus deceased patients within three categories of D-dimer levels ($n = 128$). To study if initial ADAMTS13 activity at presentation has predictive value, we selected an additional 40 cases with a sample collected within 72 hours since admission. To study the association between platelet count and ADAMTS13 activity, we selected 13 patients who had a platelet count $<70 \times 10^6$k/µL on admission. ADAMTS13, a disintegrin and metalloproteinase with a thrombospondin type 1 motif, member 13; VWF, von Willebrand factor.

 © 2021. The Author(s).

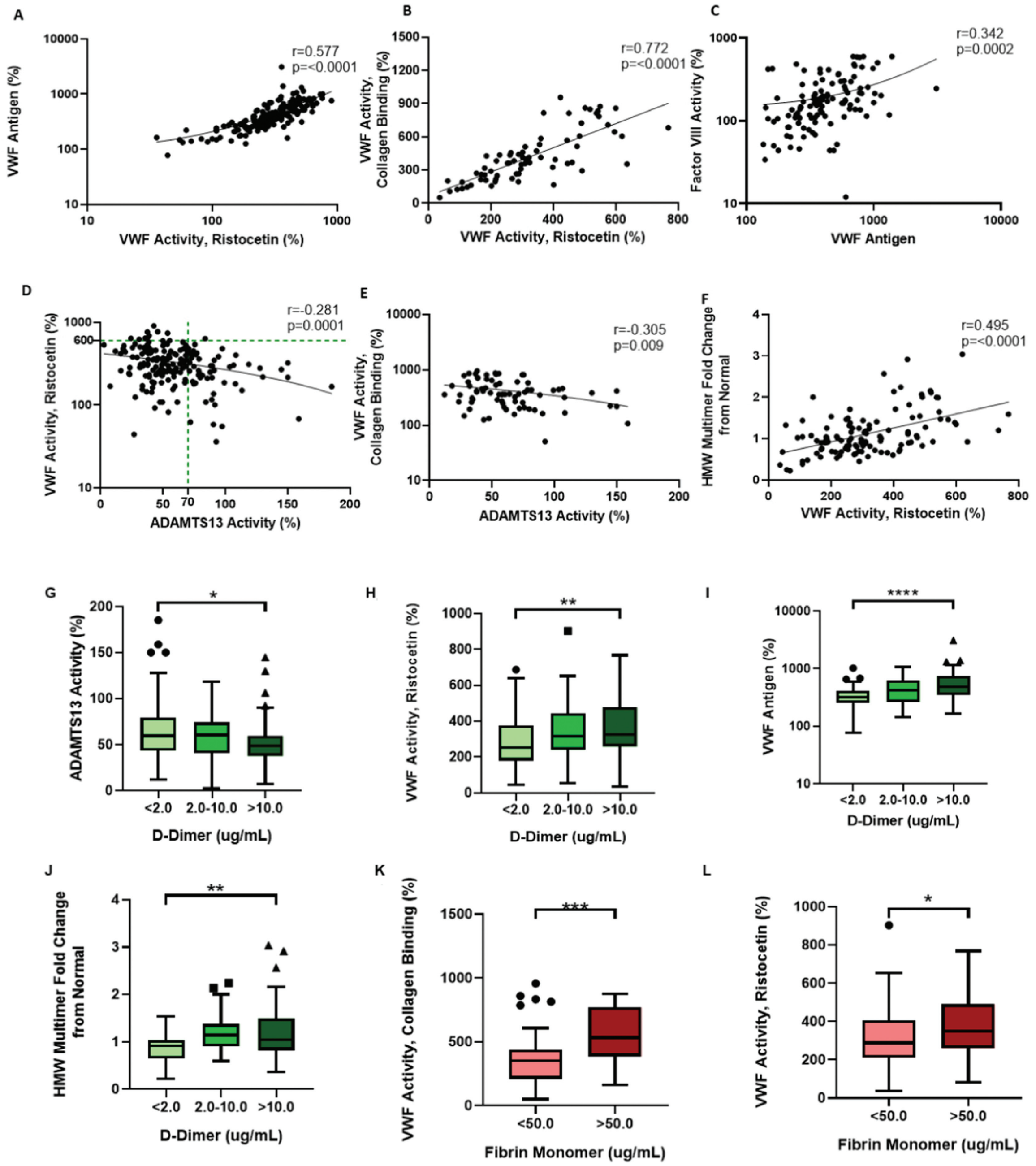

Supplementary Fig. S2 Correlations amongst VWF biomarkers and coagulation parameters. The Pearson's coefficient (r), p-value, and trendline are shown for graphs A–F. All 181 patients are represented unless otherwise stated. (**A**) Scatter plot showing positive correlation between VWF antigen and VWF Ristocetin activity. (**B**) Scatter plot showing positive correlation between VWF collagen binding activity and VWF Ristocetin activity. This only includes patients for whom an ELISA for VWF collagen binding activity was completed ($n = 72$). (**C**) Scatter plot showing slight positive correlation between factor VIII activity and VWF antigen ($n = 116$). (**D**) Scatter plot showing negative correlation between VWF Ristocetin activity and ADAMTS13 activity. Almost all (10/11) VWF activity levels greater than 600% occur in patients with ADAMTS13 activity levels less than 70%. (**E**) Scatter plot showing negative correlation between VWF collagen binding activity and ADAMTS13 activity ($n = 72$). (**F**) Scatter plot showing positive correlation between VWF Ristocetin activity and the fold change of each patient's HMW multimer size compared to the HMW multimer size of the normal pooled plasma control. This only includes patients for whom multimer western blots were ran ($n = 115$). (**G–L**) Within each box plot, the horizontal line indicates the median, the outside bars indicate the 25th and 75th percentile, individual dots indicate outlier points, and asterisk represents the p-value from a one-way ANOVA (if three values), or two tailed t-test (if two values). The asterisk indicates significance as follows: $^{*}p < 0.05$, $^{**}p < 0.01$, $^{***}p < 0.001$, and $^{****}p < 0.0001$. The box plot shows (**G**) ADAMTS13 activity ($n = 181$), (**H**) VWF Ristocetin activity ($n = 181$), (**I**) VWF Antigen ($n = 181$), (**J**) fold change in HMW multimer compared to normal ($n = 115$), stratified by low (<2 µg/mL), medium (2.0–10.0 µg/mL), or high (>10 µg/mL) D-dimer concentration. Generally, each lab parameter became more abnormal in the medium and high D-dimer stratification compared to the low stratification. The remaining box plots show (**K**) VWF collagen binding activity ($n = 72$) and (**L**) VWF Ristocetin activity ($n = 181$), stratified by low (<50 µg/mL) or high (>50 µg/mL) fibrin monomer concentration. Generally, each lab parameter was more abnormal in the high fibrin monomer stratification compared to the low stratification. ADAMTS13, a disintegrin and metalloproteinase with a thrombospondin type 1 motif, member 13; HMW, high molecular weight; VWF, von Willebrand factor.

 © 2021. The Author(s).

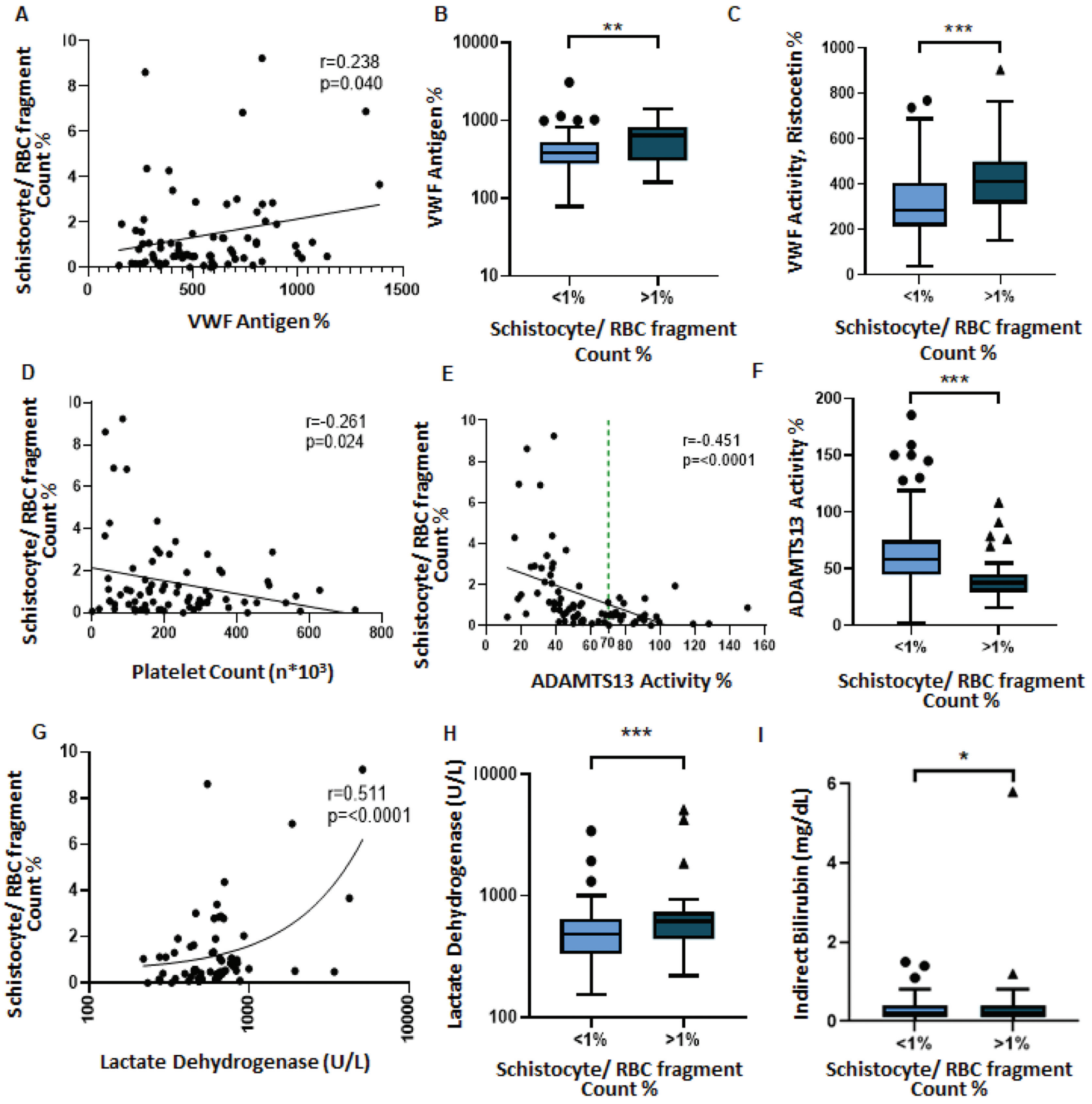

Supplementary Fig. S3 Correlation of various hemolysis, VWF biomarkers, and coagulation parameters to Schistocyte/RBC fragment count. For each scatter plot (**A**, **D**, **E G**), the Pearson's coefficient (*r*), *p*-value, and trendline are shown. Only patients for whom a CBC was flagged as abnormal within 3 days of when the sample was taken and therefore could be specifically quantified are included in the scatter plots ($n = 73$). (**A**) Scatter plot showing positive correlation between the schistocyte/RBC fragment count and VWF antigen. (**D**) Scatter plot showing negative correlation between the schistocyte/RBC fragment count and platelet count. All cases of schistocyte/RBC fragment count greater than 4% occurred in patients with a platelet count less than 200,000 k/μL. (**E**) Scatter plot showing negative correlation between the schistocyte/RBC fragment count and ADAMTS13 activity. All cases of schistocyte/RBC fragment count greater than 2% occurred in patients who had ADADMTS13 activity less than normal 70% (*dotted green line*). (**G**) Scatter plot showing positive correlation between the schistocyte/RBC fragment count and lactate dehydrogenase. For each box plot (**B**, **C**, **F**, **H**, **I**), the horizontal line indicates the median, the outside bars indicate the 25th and 75th percentile, individual dots indicate outlier points, and the asterisk represents the *p*-value from a two tailed t-test. The asterisk indicates significance as follows: $^{*}p < 0.05$, $^{**}p < 0.01$, $^{***}p < 0.001$, and $^{****}p < 0.0001$. All patients are included in the box plots ($n = 181$) unless otherwise noted, with patients without an abnormal CBC flag within 3 days of the sample assumed to have <1% schistocyte/RBC fragment count (see methods). The box plots show (**B**) VWF antigen, (**C**) VWF Ristocetin activity, (**F**) ADAMTS13 activity, (**H**) lactate dehydrogenase ($n = 158$), and (**I**) indirect bilirubin levels ($n = 171$) stratified by low (<1%) or high (>1%) schistocyte count. ADAMTS13, a disintegrin and metalloproteinase with a thrombospondin type 1 motif, member 13; CBC, complete blood count; RBC, red blood cell; VWF, von Willebrand factor.

 © 2021. The Author(s).

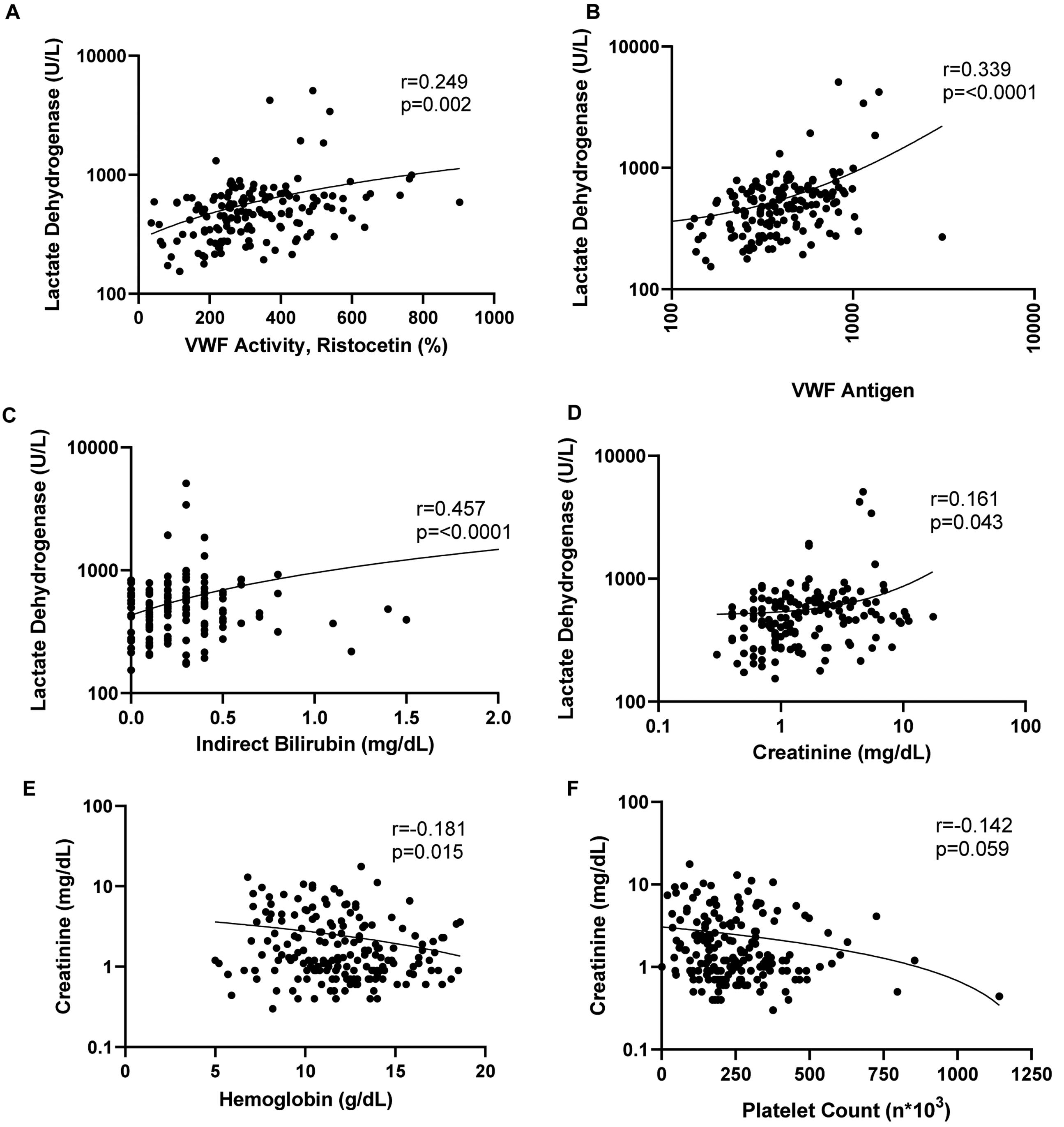

Supplementary Fig. S4 Correlations amongst various laboratory values measured to assess hemolysis and/or coagulopathies. The Pearson's coefficient (*r*), *p*-value, and trendline are shown for each graph. All 181 patients are represented in each plot unless otherwise stated. (**A**) Scatter plot showing positive correlation between LDH level and VWF Ristocetin activity ($n = 158$) (**B**) Scatter plot showing positive correlation between LDH level and VWF antigen ($n = 158$). (**C**) Scatter plot showing positive correlation between LDH level and indirect bilirubin. The only cases for which LDH and bilirubin measurements were taken within 48 hours of the sample are included ($n = 158$) (**D**) Scatter plot showing positive correlation between LDH level and creatinine level ($n = 158$) (**E**) Scatter plot showing negative correlation between creatinine level and hemoglobin level (**F**) Scatter plot showing negative correlation between creatinine level and platelet count. LDH, lactate dehydrogenase; VWF, von Willebrand factor.

 © 2021. The Author(s).

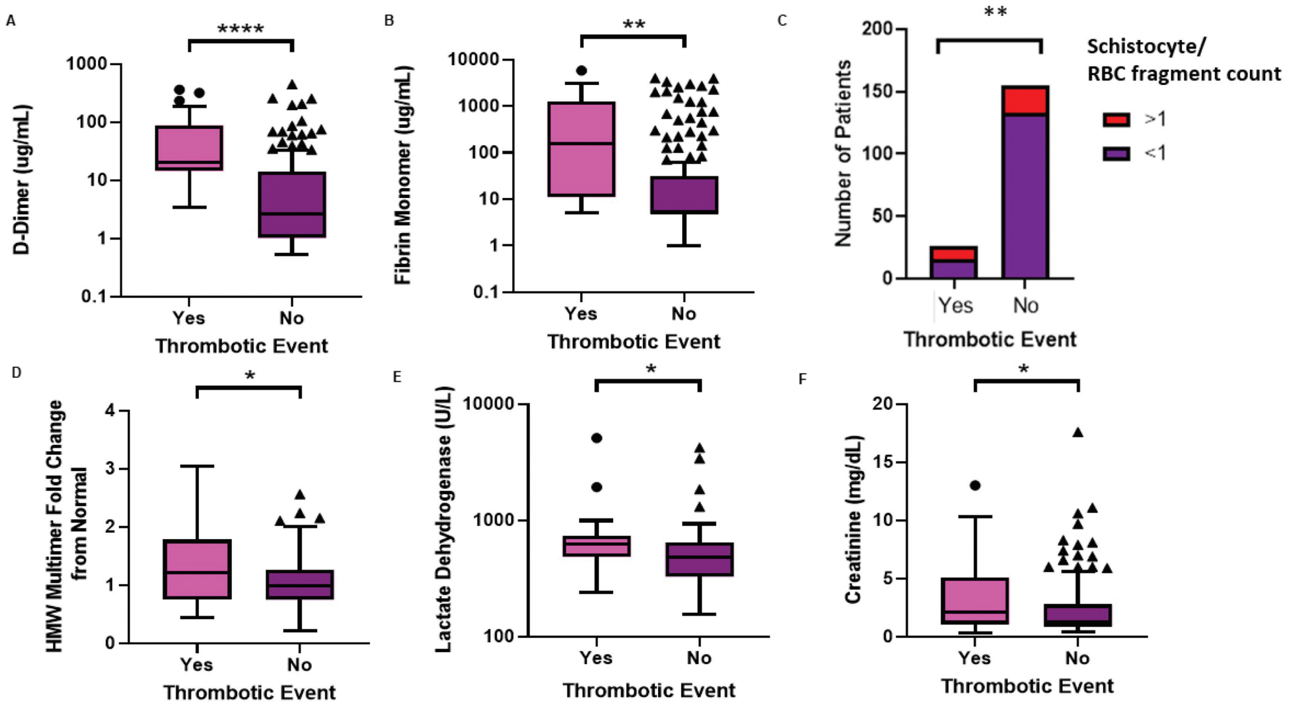

Supplementary Fig. S5 Markers of coagulation, VWF, or hemolysis stratified by the occurrence of a thrombotic event. We considered a thrombotic event to be either an occurrence of in vivo thrombosis if it was documented with radiographic imagining, or an ex vivo clot if it was reported in the patient's chart. All events within 7 days of collection of the blood sample we used to measure the markers of coagulation and hemolysis were considered. Within each box plot, the horizontal line indicates the median, the outside bars indicate the 25th and 75th percentile, individual dots indicate outlier points, and asterisk represents the *p*-value from a two tailed *t*-test. The asterisk indicates significance as follows: $^{*}p < 0.05$, $^{**}p < 0.01$, $^{***}p < 0.001$, and $^{****}p < 0.0001$. The box plots show (**A**) D-dimer, (**B**) fibrin monomer, (**C**) number of cases with schistocytes/RBC fragment >1% (*red*) versus <1% (*purple*), (**D**) fold change of HMW multimer size compared with that of normal pooled plasma, (**E**) LDH, or (**F**) creatinine level stratified by a thrombosis or clotting event within 7 days of the sample. HMW, high molecular weight; VWF, von Willebrand factor.

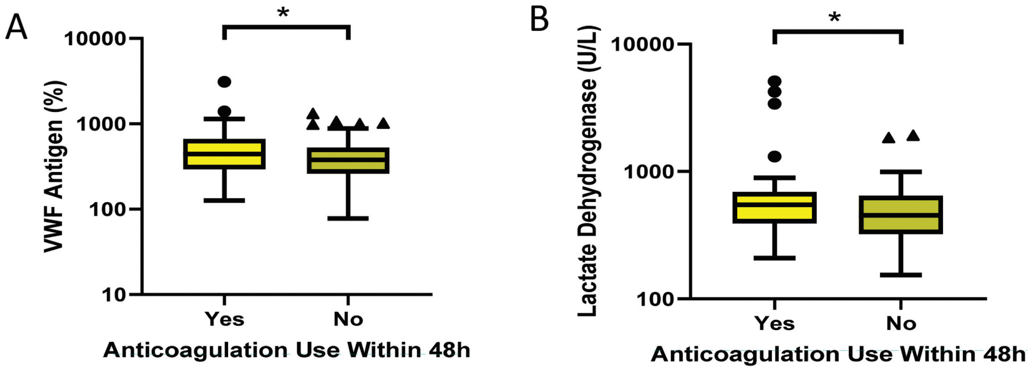

Supplementary Fig. S6 VWF antigen and lactate dehydrogenase stratified by anticoagulation use. Within each box plot, the horizontal line indicates the median, the outside bars indicate the 25th and 75th percentile, individual dots indicate outlier points, and asterisk represents the *p*-value from a two tailed *t*-test. The asterisk indicates significance as follows: $^{*}p < 0.05$, $^{**}p < 0.01$, $^{***}p < 0.001$, and $^{****}p < 0.0001$. The box plots show (**A**) VWF antigen and (**B**) lactate dehydrogenase stratified by anticoagulation use. A patient was considered to be on anticoagulation medication if it was administered at least 48 hours prior to when the sample was taken. VWF, von Willebrand factor.

 © 2021. The Author(s).

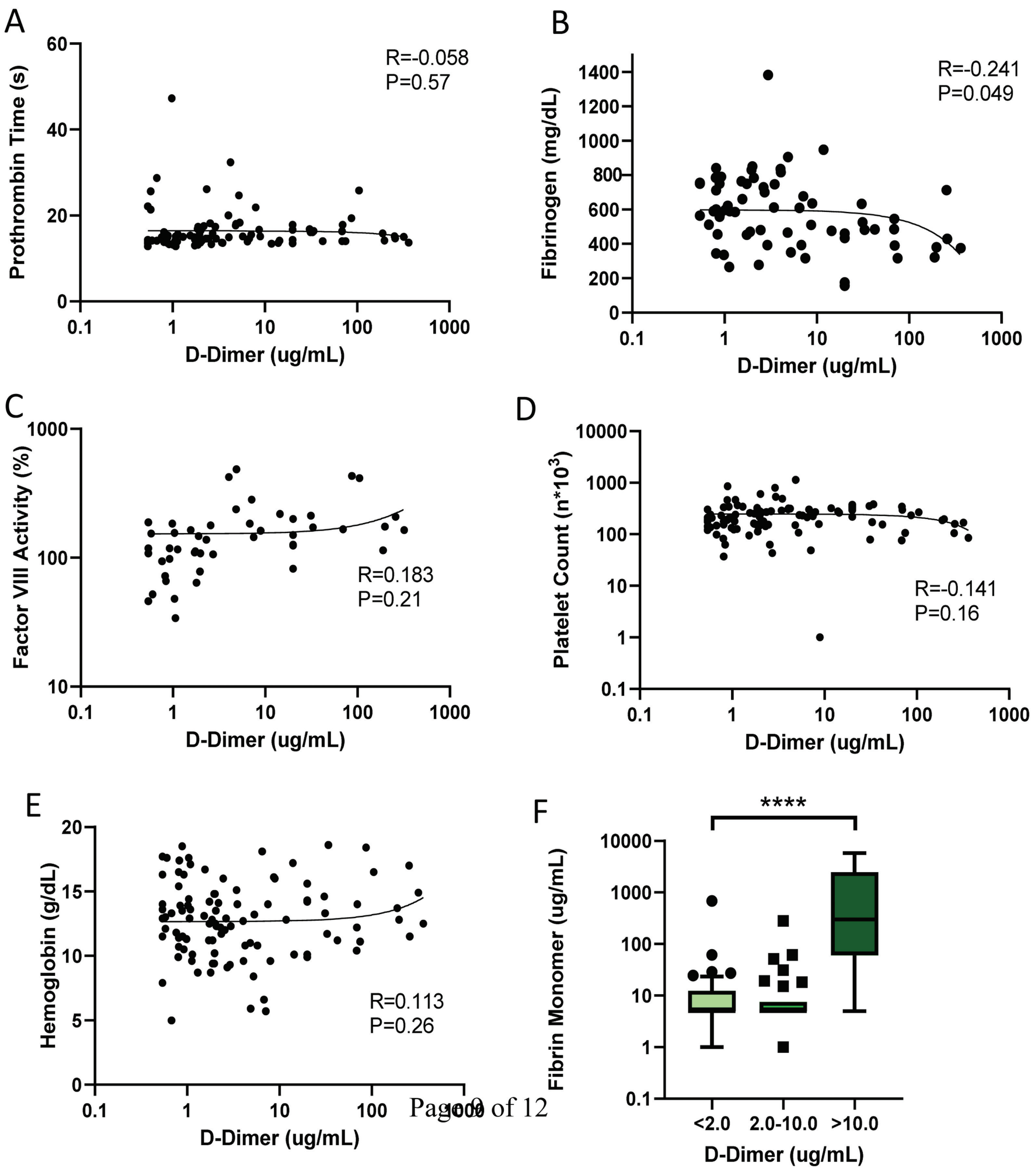

Page 9 of 12

Supplementary Fig. S7 Correlation of D-dimer with other classic markers of disseminated intravascular coagulation (DIC) within 72 hours of admission. The Pearson's coefficient (*r*), *p*-value, and trendline are shown for each graph. All 102 patients for whom an ADAMTS13 activity measurement was taken within 72 hours of admission are represented unless otherwise stated. (**A**) Scatter plot showing no significant correlation between prothrombin time and D-dimer, (**B**) Scatter plot showing slight negative correlation between fibrinogen and D-dimer ($n = 67$), (**C**) Scatter plot showing no significant correlation between factor VIII activity and D-dimer ($n = 48$), (**D**) Scatter plot showing no significant correlation between platelet count and D-dimer, (**E**) Scatter plot showing no significant correlation between hemoglobin and D-dimer, (**F**) box plot of fibrin monomer stratified by low (<2 µg/mL), medium (2.0–10.0 µg/mL), or high (>10 µg/mL) D-dimer concentration. The horizontal line indicates the median, the outside bars indicate the 25th and 75th percentile, individual dots indicate outlier points, and asterisk represents the *p*-value from a one-way ANOVA. Four asterisks indicate the *p*-value is <0.0001. ADAMTS13, a disintegrin and metalloproteinase with a thrombospondin type 1 motif, member 13; ANOVA, analysis of variance.

 © 2021. The Author(s).

Supplementary Table S1 Thrombotic events and anticoagulation treatment of 181 patients with COVID-19 stratified by ADAMTS13 activity level

Characteristics, median [IQR] or *n* (%)	Low ADAMTS13 activity (<70%) ($n=129^a$)	Normal ADAMTS13 activity (>70%) ($n=52^a$)	*p*-Value
ADAMTS13 activity (%) [70–110]	46.1 [36.7, 55.4]	88.7 [76.3, 100.2]	<0.001
Schistocyte/RBC fragment count (%) [<0.5]	1.06 [0.52, 2.54]	0.48 [0.16, 0.59]	<0.001
Age	68.0 [59.0, 78.0]	64.0 [49.8, 73.0]	0.02
Sex (male)	77 (59.7)	29 (56)	0.63
Mortality	71 (55.0)	19 (37)	0.02
Continuous renal replacement therapies use	19 (14.7)	6 (12)	0.75
Hemodialysis use	22 (17.1)	7 (14)	0.71
**Thrombotic or clotting event within entire hospital admission, *n* (%)**			
Thrombosis	23 (17.8)	13 (25)	0.37
• Deep venous thrombosis	14 (10.9)	10 (19)	0.21
• Pulmonary embolism	3 (2.3)	1 (2)	>0.99
• Arterial thrombosis	3 (2.3)	2 (4)	0.63
• Stroke	2 (1.6)	0 (0)	>0.99
Ex vivo clotting	10 (7.8)	1 (2)	0.18
**Thrombotic or clotting event within 7 days of ADAMTS13 activity measurement, *n* (%)**			
Thrombosis	16 (12.4)	4 (8)	0.44
• Deep venous thrombosis	10 (7.8)	3 (6)	0.76
• Pulmonary embolism	1 (0.8)	0 (0)	>0.99
• Arterial thrombosis	3 (2.3)	1 (2)	>0.99
• Stroke	2 (1.6)	0 (0)	>0.99
Ex vivo clotting	7 (5.4)	0 (0)	0.20
**Anticoagulation[b], *n* (%)**			
None	80 (62.1)	34 (65)	0.67
Prophylactic	26 (20.2)	12 (23)	0.81
• Heparin	9 (7.0)	1 (2)	0.29
• Enoxaparin	10 (7.8)	4 (8)	>0.99
• Apixaban	7 (5.4)	7 (14)	0.13
Therapeutic	23 (17.8)	6 (12)	0.41
• Heparin	1 (0.8)	1 (2)	0.49
• Enoxaparin	4 (3.1)	0 (0)	0.58
• Apixaban	9 (7.0)	3 (6)	>0.99
• Bivalirudin	8 (6.2)	2 (4)	0.73
• Warfarin	1 (0.8)	0 (0)	>0.99
**Anticoagulation before thrombosis or ex vivo clot[c], *n* (%)**			
None	13/30 (43)	5/13 (38.5)	>0.99
Prophylactic	4/30 (13)	4/13 (31)	0.22
Therapeutic	11/30 (37)	4/13 (31)	>0.99
Change of anticoagulation after thrombosis	19/30 (63)	11/13 (85)	0.30

Abbreviations: IQR, interquartile range; ADAMTS13, a disintegrin and metalloproteinase with a thrombospondin type 1 motif, member 13.
[a]Unless otherwise stated.
[b]Anticoagulation status within 48 hours prior to clot or ADAMTS13 measurement.
[c]Out of patients who experienced thrombosis or an ex vivo clot; 48 hours prior to clot.

 © 2021. The Author(s).
